# Socioeconomic and racial/ethnic inequalities in depression prevalence and the treatment gap in Brazil: A decomposition analysis

**DOI:** 10.1016/j.ssmph.2022.101266

**Published:** 2022-10-11

**Authors:** Matías Mrejen, Thomas Hone, Rudi Rocha

**Affiliations:** a Instituto de Estudos para Políticas de Saúde, São Paulo, Brazil; b Public Health Policy Evaluation Unit, Imperial College London, London, UK; c São Paulo School of Business Administration (FGV EAESP) & Instituto de Estudos para Políticas de Saúde, São Paulo, Brazil

## Abstract

Depression is a major global health burden and there are stark socioeconomic inequalities in both the prevalence of depression and access to treatment for depression. In Brazil, racial/ethnic inequalities are of particular concern, but the factors contributing to these inequalities remain mostly unknown. This paper firstly explores determinants of depression and the treatment gap (i.e., untreated afflicted individuals) in Brazil and identifies if socio-economic and health system factors explain changes over time. Secondly, it analyses income and racial/ethnic inequalities in depression and the treatment gap and identifies factors explaining inequalities through decomposition methods. Data from two waves (2013 and 2019) of a representative household-based survey are used. In 2019, 10.8% of adults were depressed, but over 70% of depressed adults did not receive care. Black or brown/mixed Brazilians were more likely to have untreated depression, and region of residence was the most important determinant of these racial/ethnic inequalities. Notably, 44.6% of the difference in the treatment gap between white individuals and black and brown/mixed individuals was not explained by differences in observables, which could potentially be due to discrimination or difficulties in accessing treatment due to other non-observable characteristics. Employment, age, exposure to violence and physical activity are the main contributing factors to income inequalities in depression. These results suggest that policies aimed at improving the levels of exposure of lower-income individuals to risk factors may positively impact mental health and mental health inequalities, while addressing inequalities in service provision and resourcing for mental health and tackling barriers to access stemming from discrimination are essential to bridge the treatment gap equitably.

## Highlights

• In 2019, 10.8% of adults were depressed, but over 70% of depressed adults did not receive care.
• Lower income individuals were more likely to have depression.
• Black or brown/mixed individuals were more likely to have untreated depression.
• Region of residence was the most important determinant of racial/ethnic inequalities in the treatment gap.
• Exposure to violence and physical activity are relevant contributing factors to income inequalities in depression.

## 1 Introduction

The global burden of disease attributable to mental disorders accounted for 14.6% of the years lived with disability (YLDs) globally in 2019, with more than one third due to depressive disorders (Institute for Health Metrics and Evaluation, 2021). However, providing adequate access to mental healthcare is a challenge for many health systems. On average, countries spend small shares of their health budgets on mental health services and those funds are largely spent on specialized mental health hospitals (Patel et al., 2018). In low- and middle-income countries (LMICs), between 79% and 93% of people with depression do not receive care (Esponda et al., 2020). Countries must expand services for mental health disorders, including depression, as a key step for achieving universal health coverage (World Health Organization, 2019).

Inequalities in health and access to healthcare have been widely documented in the literature (Barbosa & Cookson, 2019; França et al., 2016; Hone et al., 2017; Triaca et al., 2020), but evidence on the driving factors behind inequalities in mental health is scarcer. In particular, evidence on the driving factors behind racial/ethnic inequalities in mental health in the context of LMICs, where providing mental health care might be more challenging, is very limited. In LMICs, challenges to providing mental health care may include shortages of mental health workers, deficient research capacity, stigmatization of mental illness and separation of mental healthcare from other healthcare services (Wainberg et al., 2017). Additionally, social determinants of mental health and mental health inequalities might be particularly relevant in those contexts. Mental health is affected by demographic factors (e.g., age, sex, race/ethnicity), economic factors (e.g., income, education, employment), social factors (e.g., social support and social capital), and neighborhood characteristics (e.g., violence levels) (Lund et al., 2014, 2018); and those factors might have a more pronounced influence in poorer and more unequal societies. Lifestyle is also a determinant of mental health (Giuntella et al., 2021). Additionally, in societies where structural racism is prominent, formal and informal institutional arrangements shape the distribution of social determinants of health in prejudice of some racial/ethnic groups (Bailey et al., 2017). Therefore, quantifying the contribution of different factors to socioeconomic and racial/ethnic inequalities in mental health and access to mental healthcare is crucial to identify potential policy targets for reducing those inequalities.

In Brazil, the burden of disease attributable to mental disorders is high – 7.5% of disability-adjusted life years (DALYs) are due to mental disorders, comparable to Western Europe (7.4%) (Institute for Health Metrics and Evaluation, 2021). In 2013, 7.9% of the people aged 18 years or older had depression, however less than a quarter received any treatment (Lopes et al., 2016). Evidence suggests racial/ethnic inequalities exist in both depression prevalence and in the treatment gap for depression (i.e., untreated afflicted individuals) with individuals self-identifying as black or mixed/brown reporting higher burdens (Faisal-Cury et al., 2021; Smolen & Araújo, 2017; Stopa et al., 2015).

Brazil is an important setting for evaluating trends and inequalities in depression prevalence and in the treatment gap. On the one hand, socio-economic inequalities are large. Brazil is one of the most unequal countries in the world: the Gini Index was 53.5 in 2019, the sixth largest worldwide and the largest in Latin America (World Bank, 2021). Racial/ethnic inequalities are particularly stark, stemming from a history of slavery and structural discrimination. Brazil was one of the last countries to abolish slavery, in 1888, when approximately 30% of the population was made up of slaved individuals. However, despite all structural changes in Brazilian society, white Brazilians have been consistently over-represented at the top deciles of the income distribution since then (Assouad et al., 2018; Lima et al., 2018). Currently, brown/mixed individuals (46.8% of the total population in 2019) and black individuals (9.4%) are a majority of the population, while white individuals (42.7%) also represent a sizable share (Instituto Brasileiro de Geografia e Estat í stica, 2022). Mixed/brown and black individuals are usually grouped together when analyzing racial/ethnic inequalities in Brazil, as well as in affirmative action policies. In 2018, 32.9% of black or brown/mixed individuals earned less than US$5.5/day compared to 15.4% for white Brazilians. Illiteracy and homicide rates are also significantly higher among black or brown/mixed individuals (Instituto Brasileiro de Geografia e Estatística, 2019). More recently, between 2014 and 2016 Brazil suffered a major economic recession and has experienced unstable economic growth since then. In 2019, GDP per capita was 6.8% lower in constant prices than in 2013, and the unemployment rate grew from 7% in 2013 to 11.9% in 2019 (World Bank, 2021). During that period, increasing unemployment rates were associated with higher mortality among black or brown/mixed individuals (Hone et al., 2019).

On the other hand, Brazil operates a publicly funded national health service that provides healthcare free at the point of care. The Family Health Strategy (FHS), a community-based primary healthcare program, is the core of the health system and has been expanded since the mid-1990s. FHS currently covers over 60% of the population and expansion of FHS teams (FHTs) has been associated with improved population health (Bhalotra et al., 2020; Hone et al., 2020; Mrejen et al., 2021) and reduced health inequalities (Hone et al., 2021). In the 2000s efforts were made to increase services provision, including mental healthcare (Athié et al., 2016), although access to specialized care remains a challenge with long waiting lines (Castro et al., 2019). There is also a sizable private healthcare sector covering approximately one quarter of the population, who are mainly higher-income in urban centers (Rocha et al., 2021).

This paper has two goals. Firstly, we start by delivering a comprehensive characterization of the recent trends in the prevalence of depression and in the treatment gap in Brazil as well as in their main driving forces. It is particularly relevant to identify the underlying observable factors associated with changes in depression in light of the significant increase in prevalence between 2013 and 2019, a relatively short time frame and that coincides with a profound economic recession. Secondly, and our main goal, we identify factors explaining income and racial/ethnic inequalities in depression prevalence and in the treatment gap. We use decomposition methods, which allow not only the quantification of inequalities, but also help us explain which factors contribute to these inequalities and the magnitude of those contributions. Two methods of decomposition were used. The concentration index for depression by income was decomposed to assess the factors driving inequalities across the entire income distribution (Erreygers, 2009; Van Doorslaer et al., 2011). Differences in the treatment gap between white and brown/mixed or black individuals were decomposed using the Oaxaca-Blinder method (O'Donnell et al., 2007).

While decomposition methods have been previously used to identify factors contributing to inequalities in mental health in other contexts (Chen & Rizzo, 2010; Srivastava et al., 2021), to the best of our knowledge there are few applications to LMICs, none focused on racial/ethnic inequalities. This paper therefore contributes novel evidence on the drivers of inequalities in mental health and access to related healthcare services in LMICs through decomposition methods, with the potential to identify policy targets for reducing inequalities and improving mental health outcomes.

## 2 Materials and methods

### 2.1 Data

We use data from 2013 and 2019 waves of the National Health Survey (PNS). The PNS is a nationally representative household-based survey conducted by the Brazilian Institute of Geography and Statistics (IBGE) in partnership with the Ministry of Health. The survey is also representative for each of the five Brazilian regions and 27 states. The survey, which was conducted only in 2013 and 2019, includes socioeconomic characteristics and healthcare utilization for all members of sampled households. For a randomly selected household member (at least 18 years old in 2013 and at least 15 years old in 2019), the survey collects in-depth information on health, including self-perception of health status, lifestyle and diagnosis and treatment of chronic diseases. Microdata made available by the IBGE include all information needed to account for the sampling design, including weights adjusted for non-response rates and population projections (Instituto Brasileiro de Geografia e Estatística, 2014; Instituto Brasileiro de Geografia e Estat í stica, 2020).

The detailed individual questionnaire answered by selected household members includes the Brazilian version of the Patient Health Questionnaire (PHQ-9), a standard instrument extensively used for screening and diagnosis of depression. The PHQ-9 questionnaire asks the individual how often over the last two weeks they have been bothered by the symptoms of depression: “not at all” (score: 0), “less than half the days” (score: 1), “more than half the days” (score: 2), or “nearly every day” (score: 3). The total score for each individual is computed by summing the score for each symptom and indicates the severity of depression (0–4 none, 5–9 mild, 10–14 moderate, 15–19 moderately severe, 20–27 severe) (Kroenke et al., 2001; Levis et al., 2019). The PNS also includes information on the following questions: “Has a medical doctor or other health professional (such as a psychiatrist or a psychologist) ever diagnosed you with depression?” (Yes; no); and “Do you frequently visit a medical doctor or healthcare service for depression or only when you have a problem?” (Yes; only when I have a problem; never).

We use data on individuals’ responses to the PHQ-9 questionnaire, diagnosis, and treatment of depression. Additionally, we use data on: family income per capita, sex, race, age, highest educational level achieved, area of residence (urban/rural), state of residence, economic activity status, employment status, number of residents in the household, number of rooms in the household, if the partner lives in the same household, registration with a FHT, frequency of home visits received from any member of a FHT in the last 12 months, variables indicating previous medical diagnosis for chronic conditions except mental health, consumption of tobacco products, physical activity in the last three months, frequency of alcohol consumption, and frequency of participation in the last 12 months for each of the following activities: sport or artistic group activities, associations, volunteering, and religious services.

We also kept variables that required minor adjustments for compatibility between the two waves of the PNS because of slight differences in definition and/or answer categories: holder of any private health insurance, number of family members and number of friends the individual can count on, and characteristics of the dwelling (predominant materials in construction, sewerage system, number of toilets, number of rooms, and internet connection). The definition of variables identifying exposure to violence changed substantially between the two waves and it was not possible to make them compatible. Therefore, we only kept for 2019 variables identifying exposure in the last 12 months to different acts of psychological, physical, and sexual violence.

For additional analysis, we also obtained data from the Informatics Department of the Brazilian Ministry of Health on the number of psychologists and psychiatrists and population of each of the 27 Brazilian states to compute the rate of mental health professionals per 100,000 residents; and from IBGE on the Human Development Index for each state as a proxy for general living standards.

The entire sample of adults aged 18 or more that answered the detailed questionnaire included 60,202 individuals in 2013 and 88,531 in 2019. We dropped 12 observations with missing data on race/ethnicity (3 from 2013 and 9 from 2019) and 33 observations with missing data on household income (11 from 2013 and 22 from 2019). Our analytical sample is therefore composed of 60,188 observations from 2013 and 88,500 observations from 2019.

### 2.2 Variables

First, we computed the PHQ-9 score for all individuals. We identified all observations with a total score ≥10 as depressed (Levis et al., 2019). This cut-off is frequently used and is considered a sign of clinically relevant symptoms of depression. A study in Brazil found the PHQ-9 had a sensitivity of 72.5% and a specificity of 88.9% for diagnosing depression using this cut-off (Santos et al., 2013). All PHQ-9 depressed individuals that had either never been diagnosed with depression by a healthcare professional or that were diagnosed but never visited a healthcare service for depression were identified as having untreated depression – i.e., falling in the treatment gap (Evans-Lacko et al., 2018; Kohn et al., 2004, 2018). We also provide supplementary analyses grouping individuals with a PHQ-9 score ≥10 or currently receiving treatment (frequently or when having a problem).

Second, we included covariates and grouped them into five blocks of contributing factors for the decomposition analysis: socio-demographic, socio-economic, social support, healthcare services, and lifestyle and physical health. The choice of this contributing factors was based on the literature (Brunoni et al., 2020; China, 2020; de Oliveira et al., 2018; Freeman et al., 2016; Gariépy et al., 2016; Graham et al., 2007; Lorant et al., 2007; Ribeiro et al., 2009; Roy & Lloyd, 2012; Tomita et al., 2017).

Socio-demographic covariates included in analyses were: sex, age (18–24 years, 25–34, 35–44, 45–54, 55–64, 65 or more), education (none, incomplete elementary, complete elementary school, incomplete secondary, complete secondary, incomplete higher, complete higher), race/ethnicity (white, black, Asian, mixed/brown, indigenous), urban residence (yes/no) and region of residence (North, North-East, Center-West, South-East, South). Socio-economic covariates were: employment status (inactive, unemployed, employed), slum residence (a binary variable based on the UN-Habitat definition of slums (Pitcairn et al., 2021)), internet connection in the household, and income. Three income variables were used alternatively in different analysis: total income per capita in the household, income quintile (a variable indicating the quintile in the income distribution according to total income per capita in the household), and the log of family income per capita (adjusted for inflation between waves). Social support covariates were: a binary variable indicating support from any family member and/or friend, cohabitation (alone, with partner, with other person), and participation in any group, social and/or community activities (never, less than monthly, at least once per month). Healthcare covariates were: holder of a private health insurance (binary) and FHT registration (No/Does not know, Yes and did not receive any home visit from a FHT member in the last 12 months, Yes and had at least once home visit). Covariates related to lifestyle and physical health were: medical diagnosis of at least one non-mental NCD, smoking, physical activity (no, less than weekly, once or twice a week, three or more times per week), alcohol consumption (never, less than weekly, once a week, twice or more per week) and exposure to an episode of psychological, physical or sexual violence in the previous 12 months (2019 only).

### 2.3 Statistical analysis

We start by characterizing the recent trends in the prevalence of depression and in the treatment gap in Brazil as well as by characterizing socioeconomic and racial inequalities. We then use decomposition methods, which allow us to explain which factors contribute to these inequalities and the magnitude of those contributions. Two methods of decomposition were used. The decomposition of the concentration index, which is suitable to decompose inequalities across the distribution of a continuous variable, was used to assess the factors driving inequalities across the entire income distribution (Erreygers, 2009; Van Doorslaer et al., 2011). Differences in the treatment gap between white and brown/mixed or black individuals were decomposed using the Oaxaca-Blinder method, which is suitable for decomposing differences across groups defined by a binary variable (O'Donnell et al., 2007). All analyses employed survey weights to account for the sampling design of the PNS.

#### 2.3.1 Descriptive and regression analysis

First, we computed the prevalence of depression and the treatment gap for depression in 2013 and 2019 for the entire sample and by income quintile and race/ethnicity. We calculated covariate means for both years. While we focus on a relatively short time frame between PNS waves, we do observe a significant growth in the prevalence of depression in Brazil. The time frame of analysis also coincides with a profound economic recession, which therefore generated variation in socioeconomic conditions throughout the period. Second, we used linear probability models to assess associations between covariates and both depression and the treatment gap using the following form:

$$H_{g}=X\beta_{g}+\varepsilon_{g}$$

Where *H* is the outcome (being depressed or being untreated, conditional on being depressed), X is a matrix of covariates (described above) and includes a constant term, β is a vector of coefficients and ε is an error term with conditional mean zero. The subscript *g* indicates that the observation belongs to a certain group (for example, year of the PNS or race/ethnicity). The models were repeated separately for depression and for the treatment gap for *g = 2013* and for *g = 2019*.

#### 2.3.2 The concentration index and its decomposition

To assess income inequalities in depression, we plotted the concentration curve for the prevalence of depression in 2019 and computed the associated Erreygers-corrected concentration index, which measures inequalities in the prevalence of depression across the income distribution (Erreygers, 2009; Van Doorslaer et al., 2011). We plotted the concentration curve and computed the concentration index according to the individual ranking in a continuous variable measuring total household income per capita.

Concentration curves plot the cumulative percentage of a health variable against the cumulative percentage of the population ranked by living standards. The concentration index summarizes the information depicted in a concentration curve. It can be computed as:

$$C\left(h\right)=\frac{1}{n}\sum_{i=1}^{n}\left[\left(\frac{h_{i}}{\bar{h}}\right)\left(2R_{i}^{y}-1\right)\right]$$

Where C(h) is the concentration index of variable h (depression or falling in the treatment gap), $h_{i}$ is the value of h for individual i, $\bar{h}$ is the mean of h in the sample, n is the sample size, and $R_{i}^{y}=n^{-1}\left(i-0.5\right)$ is the fractional rank of individual i ordering the sample according to family income per capita (y) from the lowest to the highest value. Negative values signal higher concentrations of variable h in the poorest half of the population and positive values in the richest half (Van Doorslaer et al., 2011). If h is a bounded variable, the bounds of the concentration index depend on $\bar{h}$ (Erreygers, 2009). We therefore used a the Erreygers-corrected concentration index (Erreygers, 2009), which solves this problem and can be computed as:

$$E\left(h\right)=\left(\frac{4\bar{h}}{h_{\max}-h_{\min}}\right)C\left(h\right)$$

It is possible to decompose the concentration index to measure the contribution of individual factors to income-related inequalities in health (Van Doorslaer et al., 2011). Thus, it is possible to examine what proportion of inequality in the prevalence of depression is associated with different observable characteristics – e.g., education or exposure to violence. Supposing a linear model of the following form linking h to observed contributing factors:

$$\frac{h_{i}-h_{\min}}{h_{\max}-h_{\min}}=\beta_{0}+\sum_{j=1}^{K}\beta_{j}x_{ji}+e_{i}$$

The Erreygers-corrected concentration index can be written as:

$$E\left(h\right)=4\left[\sum_{j=1}^{K}\beta_{j}GC\left(x_{j}\right)+GC\left(e_{i}\right)\right]$$

Thus, the contribution of each factor $x_{j}$ is given by the product of the sensitivity of health with respect to that factor, the parameter $\beta_{j}\text{,}$ and the degree of income-related inequality in the distribution of that factor $GC\left(x_{j}\right)=\bar{x_{j}}\times C\left(x_{j}\right)\text{.}$ We used this decomposition technique to assess the factors contributing to socio-economic inequalities in the prevalence of depression in 2019. Covariates $x_{j}$ included were the same ones used in the regression analysis described above, but with two differences: income quintile was used as the income variable and the variable indicating exposure to violent episodes was added. The inclusion of income quintile as a covariate in the decomposition is justified for avoiding any potential omitted variable bias were it not included in the regression. All coefficients $\beta_{j}$ were estimated using a Linear Probability Model.

#### 2.3.3 Oaxaca blinder decomposition

The Oaxaca-Blinder technique decomposes differences in the mean of an outcome between two groups (O'Donnell et al., 2007). It is widely used to analyze factors associated with differences in health outcomes or access to healthcare between two groups or their evolution between two different points in time (Brydsten et al., 2018; Brzezinski, 2019).

Assuming that the probability of being depressed or being untreated (conditional on being depressed) can be explained through linear models as the one described above, letting $D_{B}$ be a binary variable that indicates belonging to group B (e.g., self-identifying as black or brown/mixed), and taking the expectations over X, the mean difference between individuals belonging to group B and individuals belonging to another group A (e.g., self-identifying as white) can be expressed as:

$$\Delta_{\bar{H}}=E\left[H_{B}|D_{B}=1\right]-E\left[H_{A}|D_{B}=0\right]$$

$$=E\left[X|D_{B}=1\right]\beta_{B}-E\left[X|D_{B}=0\right]\;\beta_{A}$$

To perform a decomposition, a counterfactual is needed. It is possible to adopt the prevalence of depression or the depression treatment gap that would have been observed in the sample of individuals belonging to group B if the coefficients linking individual characteristics to those variables would have been equal to the coefficients in the sample of individuals belonging to group A –i.e., $\left[X|D_{B}=1\right]\beta_{A}$. For example, the treatment gap that would have been observed in the subsample of depressed black or brown/mixed individuals if the coefficients linking individual characteristics to being untreated were the same as in the subsample of depressed white individuals can be used as a counterfactual. Adding and subtracting that counterfactual and replacing the expected values of the covariates by the sample averages ${\bar{X}}_{g}$, the decomposition can be estimated as:

$$\Delta_{\bar{H}}={\bar{X}}_{B}\left(\hat{\beta_{B}}-\hat{\beta_{A}}\right)+\left({\bar{X}}_{B}-{\bar{X}}_{A}\right)\hat{\beta_{A}}=\Delta_{\bar{H}}^{U}+\Delta_{\bar{H}}^{E}$$

The first term in the decomposition equation, $\Delta_{\bar{H}}^{U}$, is the “unexplained” part of the difference in the means. The second term, $\Delta_{\bar{H}}^{E}$, is the “explained” part of the difference. So, the Oaxaca-Blinder decomposition allows to analyze which part of the differences in the depression treatment gap is linked to mean characteristics of individuals according to group belonging – e.g., differences in observable characteristics between depressed black or brown/mixed and white individuals – and what part is attributable to differences in the coefficients that link those characteristics to falling in the treatment gap. Additionally, it is possible to compute the detailed decomposition to estimate the contribution of the $k^{th}$ covariate to the explained and unexplained component:

$$\Delta_{\bar{H}}^{U}=\left(\hat{\beta_{B;0}}-\hat{\beta_{A;0}}\right)+\sum_{k=1}^{M}{\bar{X}}_{B;k}\left(\hat{\beta_{B;k}}-\hat{\beta_{A;k}}\right)$$

$$\Delta_{\bar{H}}^{E}=\sum_{k=1}^{M}\left({\bar{X}}_{B;k}-{\bar{X}}_{A;k}\right)\hat{\beta_{A;k}}$$

We estimated these decompositions to analyze factors associated with differences in the treatment gap for depression between white individuals (group A) and mixed/brown or black individuals (group B) in 2019. Mixed/brown and black individuals were grouped as is common practice when analyzing racial/ethnic inequalities in Brazil (Instituto Brasileiro de Geografia e Estatística, 2019). For the analysis of racial/ethnic differences, linear probability models were estimated as described above for regression analysis, but with two differences: the income quintile was used as the income variable and a variable indicating if the individual had suffered any violent episode in the previous 12 months was added.

In supplementary analysis, Oaxaca-Blinder decompositions were used to analyze the contributing factors to the evolution of the prevalence of depression and the treatment gap for depression between 2013 and 2019 using the coefficients obtained from the regressions described above.

The inclusion of categorical covariates with more than two categories generates challenges for the interpretation of individual contributions. For the “explained” part, the contribution of each individual category varies with the choice of the omitted group, but the contribution of the categorical variable as a whole remains unchanged. However, for the unexplained part, changing the base category also changes the contribution of the categorical variable as a whole (Fortin et al., 2011). We therefore restrict our analysis of detailed contributions of each factor to the explained part of the decomposition, focusing on the contribution of each variable as a whole rather than on the contribution of each specific category.

## 3 Results

### 3.1 Descriptive and regression analysis

The prevalence of depression among the population aged 18 or older increased 2.9 percentage points (p.p.) from 7.9% in 2013 to 10.8% in 2019 when considering our main definition for depression – i.e., PHQ9 score ≥10 (Table 1). The treatment gap (untreated individuals with depression) decreased from 76.1% to 71.2%. In the same period, the share of the population currently treated for depression grew from 5% to 7.2%. Grouping individuals with depressive symptoms together with those currently receiving treatment, we also see an increase in the share of affected individuals (from 11% in 2013 to 14.9% in 2019) and a fall in the share of those individuals that are not receiving any treatment (from 54.6% to 51.6%). Additionally, particularly relevant for our analysis are the patterns of increasing self-identification of black and brown/mixed, higher unemployment rates, stagnant income and higher coverage of primary healthcare services.

**Table 1:** Summary statistics.

Variable	2013	2019	Difference	
	(n = 60,188)	(n = 88,500)		
	Mean	Mean	Difference	p-value
**Depression (PHQ9 ≥ 10)**	0.079	0.108	0.029	≤0.001
**Treatment gap**^**a**^	0.761	0.712	−0.049	≤0.001
**Currently treated**	0.050	0.072	0.022	≤0.001
**Depression (PHQ9 ≥ 10) or Currently treated**	0.110	0.149	0.039	≤0.001
**Treatment gap (PHQ9 ≥ 10 or Currently treated)**^**b**^	0.546	0.516	−0.03	0.013
**Woman**	0.529	0.532	0.003	≤0.001
**Age**				
18–24	0.159	0.139	−0.02	≤0.001
25–34	0.217	0.181	−0.036	≤0.001
35–44	0.192	0.202	0.01	0.001
45–54	0.175	0.178	0.003	0.229
55–64	0.134	0.150	0.016	≤0.001
65 or older	0.123	0.149	0.026	≤0.001
**Race**				
White	0.476	0.433	−0.043	≤0.001
Black	0.091	0.115	0.024	≤0.001
Asian	0.009	0.009	0	0.860
Browns/Mixed	0.420	0.438	0.018	≤0.001
Indigenous	0.004	0.005	0.001	0.038
**Education**				
None	0.137	0.061	−0.076	≤0.001
Basic incomplete	0.253	0.287	0.034	≤0.001
Basic complete	0.099	0.078	−0.021	≤0.001
Secondary incomplete	0.056	0.067	0.011	≤0.001
Secondary complete	0.281	0.298	0.017	≤0.001
Higher incomplete	0.047	0.051	0.004	0.043
Higher complete	0.127	0.158	0.031	≤0.001
**Urban**	0.862	0.862	0	0.998
**Region**				
North	0.075	0.078	0.003	≤0.001
North-East	0.265	0.265	0	≤0.001
South-East	0.439	0.434	−0.005	≤0.001
South	0.148	0.147	−0.001	≤0.001
Center-West	0.074	0.076	0.002	≤0.001
**Slum proxy**	0.188	0.151	−0.037	≤0.001
**Internet**	0.489	0.846	0.357	≤0.001
**Employment status**				
Inactive	0.351	0.335	−0.016	≤0.001
Unemployed	0.034	0.053	0.019	≤0.001
Employed	0.615	0.613	−0.002	0.597
**Log family income per capita (R$ 2019)**	6.857	6.836	−0.021	0.126
**Support of family and/or friends**	0.935	0.981	0.046	≤0.001
**Lives with**				
Alone	0.067	0.075	0.008	≤0.001
Partner	0.613	0.614	0.001	0.790
Other person	0.320	0.311	−0.009	0.029
**Participation in group, social and/or community activities**				
Never	0.224	0.167	−0.057	≤0.001
Less than monthly	0.198	0.189	−0.009	0.017
At least once per month	0.577	0.644	0.067	≤0.001
**Health insurance**	0.302	0.296	−0.006	0.383
**Registered with Family Health Team**				
No/Does not know	0.455	0.385	−0.07	≤0.001
Yes, and no home visits in last 12 months	0.096	0.144	0.048	≤0.001
Yes, and at least one home visit in last 12 months	0.448	0.471	0.023	0.006
**Diagnosis of non-mental NCD**	0.486	0.544	0.058	≤0.001
**Tobacco**	0.146	0.126	−0.02	≤0.001
**Physical activity**				
No	0.685	0.580	−0.105	≤0.001
Yes, less than weekly	0.011	0.015	0.004	≤0.001
Yes, once or twice a week	0.117	0.147	0.03	≤0.001
Yes, three or more times per week	0.187	0.258	0.071	≤0.001
**Alcohol**				
Never	0.596	0.578	−0.018	≤0.001
Yes, less than weekly	0.164	0.158	−0.006	0.063
Yes, once a week	0.108	0.118	0.01	0.002
Yes, twice or more per week	0.131	0.146	0.015	≤0.001

Note: the table shows means intervals for all variables used in the decomposition analysis of the evolution of the prevalence of depression and the treatment gap for depression, as well as the difference in means and the corresponding two-sided p-value. All reported data are weighted considering the sampling design. ^a^ Data on the treatment gap are only referent to the subsample of depressed (PHQ9 ≥ 10) individuals – complete summary statistics for this subsample are presented in Table A1 in the Appendix. ^b^ Data are only referent to the subsample of depressed (PHQ9 ≥ 10) or currently treated individuals.

**Fig. 1:**
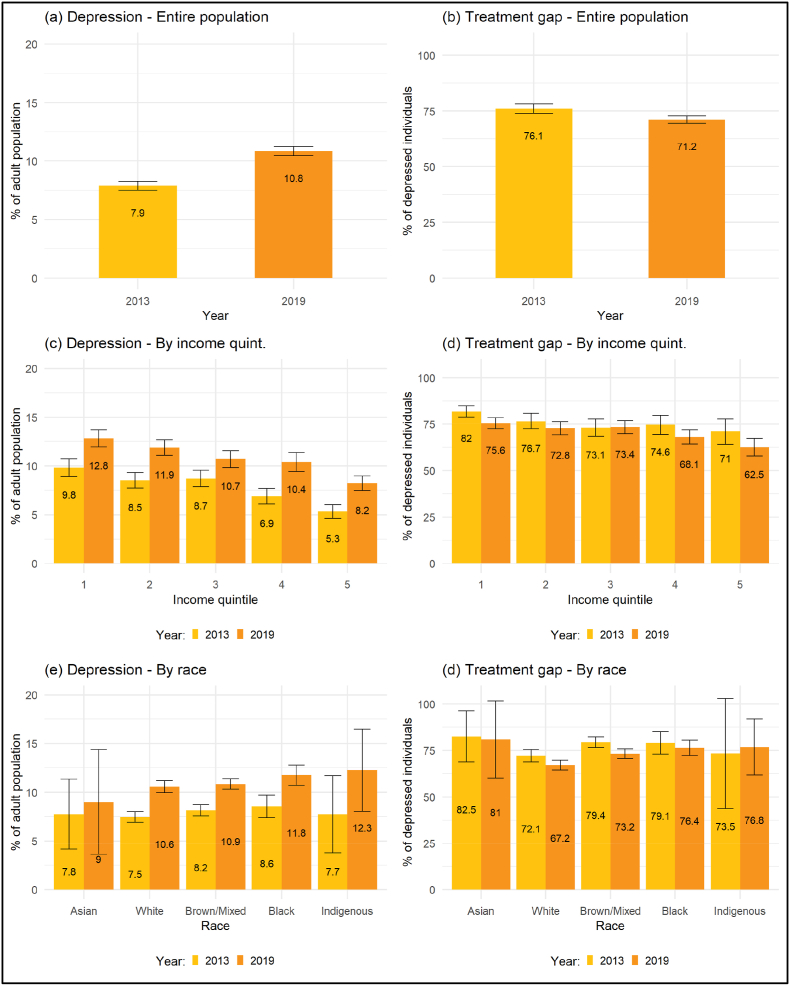
Prevalence of Depression (PHQ9 ≥ 10) and Treatment Gap, 2013–2019 (Entire population, by income quintile and by race) Note: the figure shows the evolution of the prevalence of depression (PHQ9 ≥ 10, panel a) and the treatment gap for depression (panel b) in Brazil in 2013 and 2019. Panels c and d show the same results, but according to income quintile, and panels e and f according to racial/ethnic self-identification. The error bars display the 95% confidence intervals. All reported data are weighted considering the sampling design.

Fig. 1 shows the evolution of depression prevalence and the treatment gap for depression between 2013 and 2019 by income quintile and race/ethnicity. The prevalence of depression increased across all quintiles of the income distribution, but proportionally more among higher-income individuals than among poorer individuals. For example, it increased 54.7% in the fifth (richest) quintile – from 5.3% (95%CI: 3.6%–6%) in 2013 to 8.2 (95%CI: 7.5%–9%) in 2019 – and 30.6% in the first (poorest) quintile – from 9.8% (95% CI: 8.9%–10.7%) in 2013 to 12.8% (95%CI: 12%–13.7%) in 2019. Changes in the treatment gap over time were not statistically significant for any income quintile. Notably, the socio-economic gradient in depression prevalence is greater than the gradient in the treatment gap. In 2019, the difference in the prevalence of depression between the highest and lowest income quintiles was 56% compared to 20.8% for the treatment gap.

Figure A1 in the Appendix shows supplementary results grouping individuals with a PHQ-9 score ≥10 or currently receiving treatment. When grouping people with depressive symptoms and people currently under treatment, the socioeconomic gradient in the treatment gap increases while the gradient in depression is attenuated to the point of any statistically significant differences between the first and fifth income quintile are detected. As probabilities of accessing treatment for depression are unequally distributed, this happens mechanically because this measure counts individuals being treated (which are more concentrated in the higher-income quintiles) as affected by depression, but by definition they do not fall in the treatment gap.

The prevalence of depression increased among white, mixed/brown and black Brazilians between 2013 and 2019. Differences between these groups were not statistically significant in any wave. In 2019, for example, prevalence of depression was 10.6% (95%CI: 10%–11.2%) among white individuals, 10.9% (95%CI: 10.3%–11.4%) among mixed/brown individuals and 11.8% (95%CI: 10.7%–12.8%) among black individuals. Reductions in the treatment gap between 2013 and 2019 were only significant among brown/mixed individuals: from 79.4% (95%CI: 76.6%–82.2%) in 2013 to 73.2% (95%CI: 70.7%–75.8%) in 2019. While still large among all groups according to racial/ethnicity self-identification in 2019, the treatment gap was significantly smaller among white Brazilians (67.2%, 95%CI: 65.5%–69.8%) than among black (76.4%, 95%CI: 72.3%–80.5%) and brown/mixed individuals (73.2%, 95%CI: 70.7%–75.8%). In all cases, the values among Asian and indigenous are too imprecisely estimated due to small numbers. Results grouping individuals with a PHQ-9 score ≥10 or currently receiving treatment show an even larger difference in the treatment gap between white and black and brown/mixed Brazilians (Figure A1 in the Appendix).

**Fig. 2:**
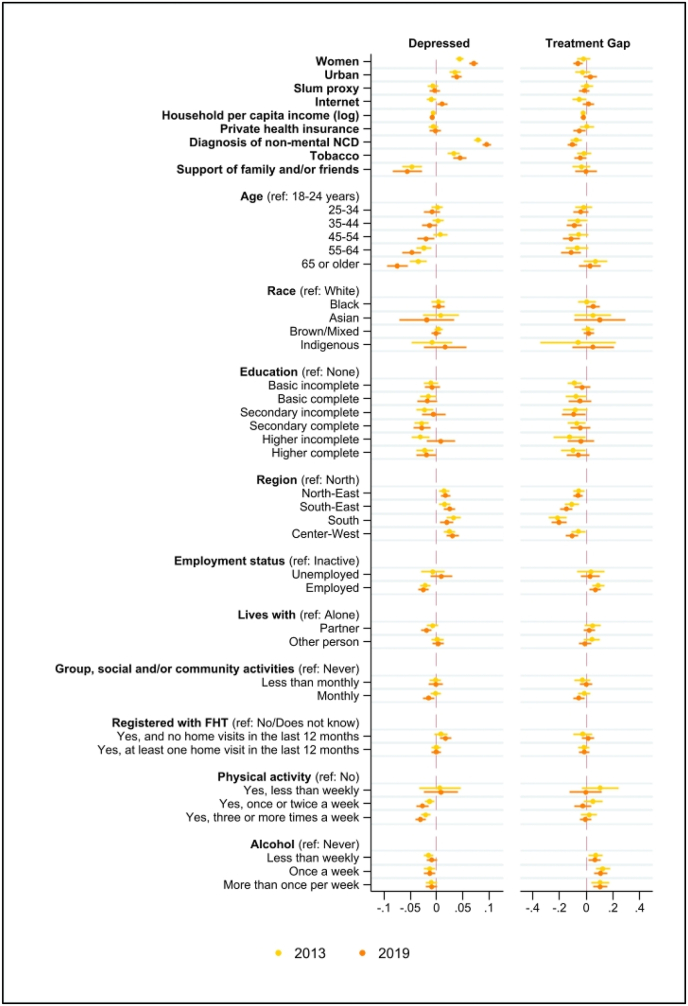
Linear probability model: depression (PHQ9 ≥ 10) and treatment gap for depression, 2013 and 2019 Note: the figure plots the coefficients from four independent linear probability models linking covariates with the probability of being depressed (PHQ9 ≥ 10) and of falling in the treatment gap –conditional on being depressed– in 2013 and in 2019. Full results are available in Table A2 in the Appendix. Coefficients represent the marginal changes in the probability of being depressed or falling in the treatment gap associated with a unitary increase in the covariate, holding constant all other regressors. All reported data are weighted considering the sampling design.

In adjusted regression models, there were key demographic, socioeconomic and health service variables associated with the depression (PHQ9 ≥ 10) or the treatment gap (Fig. 2; Table A2 in the Appendix). For example, women were 7.1 percentage points more likely to be depressed than men, holding all other variables constant (β = 0.071, 95%CI: 0.063–0.079). Other factors positively associated with the probability of being depressed in 2019 were a non-mental NCD diagnosis (β = 0.096, 95%CI: 0.087–0.104), living in an urban area (β = 0.039, 95%CI: 0.029–0.048), and smoking tobacco (β = 0.045, 95%CI: 0.033–0.057). Factors with the largest negative association with depression in 2019 were having support from family members and/or friends (β = −0.056, 95%CI: −0.084 to −0.028), being 55 or older, and exercising more than once per week. Predicted probabilities for depression and the treatment gap for all categories of categorical variables, using results from Table 1, are depicted in Table A3 in the Appendix. Predicted probabilities for the prevalence of depression do not show any significant difference between white individuals and brown/mixed or black individuals. Also, when combining income quintile and ethnicity/race, we do not see any significant difference in the predicted probabilities of depression by ethnicity/race (Figure A2 in the Appendix).

Region of residence was the factor most strongly associated with the treatment gap for depression. Compared to individuals in the North, those in the South and South-East were respectively 20.4% (β = −0.204, 95%CI: −0.259 to −0.149) and 14.8% (β = −0.148, 95%CI: −0.194 to −0.102) less likely to be untreated if depressed –and those coefficients were significantly larger in magnitude than for the Northeast region. Region fixed-effects account for all non-observable factors that vary across regions and might affect mental health or access to mental healthcare (e.g., urban infrastructure, general living standards in the place of residence or non-observable markers of availability of mental healthcare workers and services). In short, region fixed-effects indicate whether non-observable factors might be relevant in our empirical setting, and the extent to which both policymaking and future research should focus on specific regions.

In supplementary analysis, the results reported in Table 1 and Fig. 2 were tested in Oaxaca-Blinder decomposition analyses to identify factors explaining trends in the prevalence of depression between 2013 and 2019. Notably, changes in observable characteristics between 2013 and 2019 could not account for changes in depression prevalence or the treatment gap (Figure A3 and Table A4 in the Appendix).

### 3.2 Decomposition analysis

#### 3.2.1 Decomposition of socioeconomic inequalities in depression prevalence

**Fig. 3:**
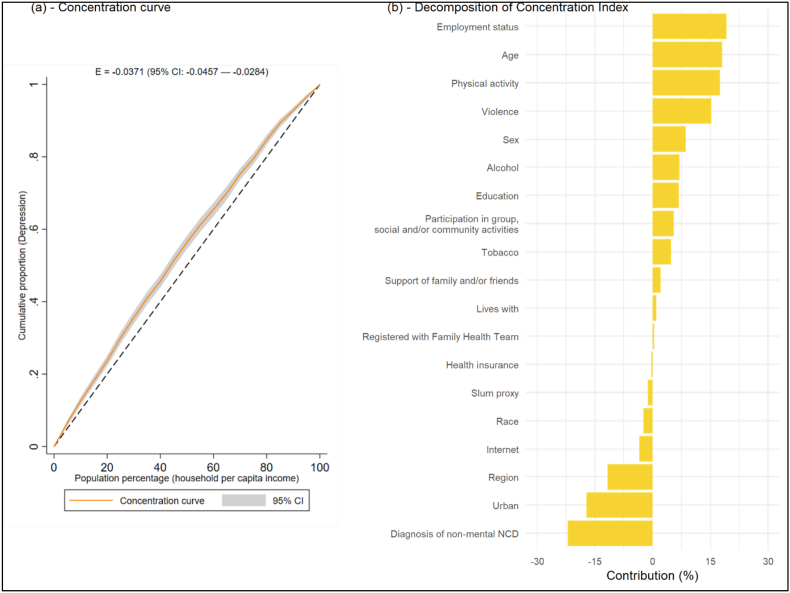
Concentration Curve and decomposition of the Erreygers-corrected concentration index for Depression (PHQ9 ≥ 10) Note: the figure shows the concentration curve and the Erreygers-corrected concentration index for the prevalence of depression (PHQ9 ≥ 10) in 2019 (panel a) and results from the decomposition of the concentration index (panel b). A variable indicating income quintile according to per capita household income was included in the analysis to avoid potential omitted variable bias, but results were excluded from the plot in panel b. Full results are available in Table A5 in the Appendix. All reported data are weighted considering the sampling design.

Income inequalities in the depression (PHQ9 ≥ 10) prevalence in 2019 were assessed across the full distribution of household income per capita (Fig. 3, panel a). The concentration curve lies above the 45° line and the associated Erreygers-corrected concentration index (E) is negative (−0.0371, 95%CI: −0.0457 to −0.0284), both indicating depression is more prevalent among poorer individuals. Decomposition of this concentration index (Fig. 3, panel b; full results in Table A5 in the Appendix) revealed the main contributors to income inequalities in depression were employment status (accounting for 19.2% of income inequalities in depression), age (18.1%), exposure to violent episodes (15.3%) and physical activity (17.5%). These factors are notably associated with income (e.g., employment, age, physical activity, and protection from violence are concentrated among higher-income individuals). Living in an urban area (−17.2%) and a non-mental NCD diagnosis (−22%) were factors identified as contributing to reductions in income inequalities in depression. Results excluding violence as a covariate suggests that the contribution of age would be overestimated, as exposure to violence disproportionally affects younger individuals (Figure A4 in the Appendix).

Figure A5 in the Appendix shows results without including region of residence in the analysis, which was considered in our main analysis to control for factors that vary across regions and could affect mental health or access to mental healthcare but are not directly observable in our data. The general picture depicted by this extension analysis is similar, but the contributions of individual factors are marginally larger, as they absorb the variation due to regional differences in our main analysis. Figure A6 in the Appendix shows results from exercises in which we replace region of residence dummies by the number of mental health professionals (psychologists plus psychiatrists) per 100,000 residents and the Human Development Index in the state of residence. Results suggest that geographical differences in the general standard of living, as measured by the HDI, are not as relevant as a contributor to socioeconomic inequalities in the prevalence of depression as other aggregate factors that vary at the regional level.

As mentioned above, using an alternative definition that groups individuals with depression measured by a PHQ-9 score ≥10 or currently treated, the socioeconomic gradient is attenuated, but the gradient in the treatment gap increases. Decomposition of the concentration index for the treatment gap using this alternative definition shows that region of residence and having private health insurance are the two largest contributors to socioeconomic inequalities in the treatment gap (Figure A7 in the Appendix).

#### 3.2.2 Decomposition of racial/ethnic inequalities in the treatment gap for depression

**Fig. 4:**
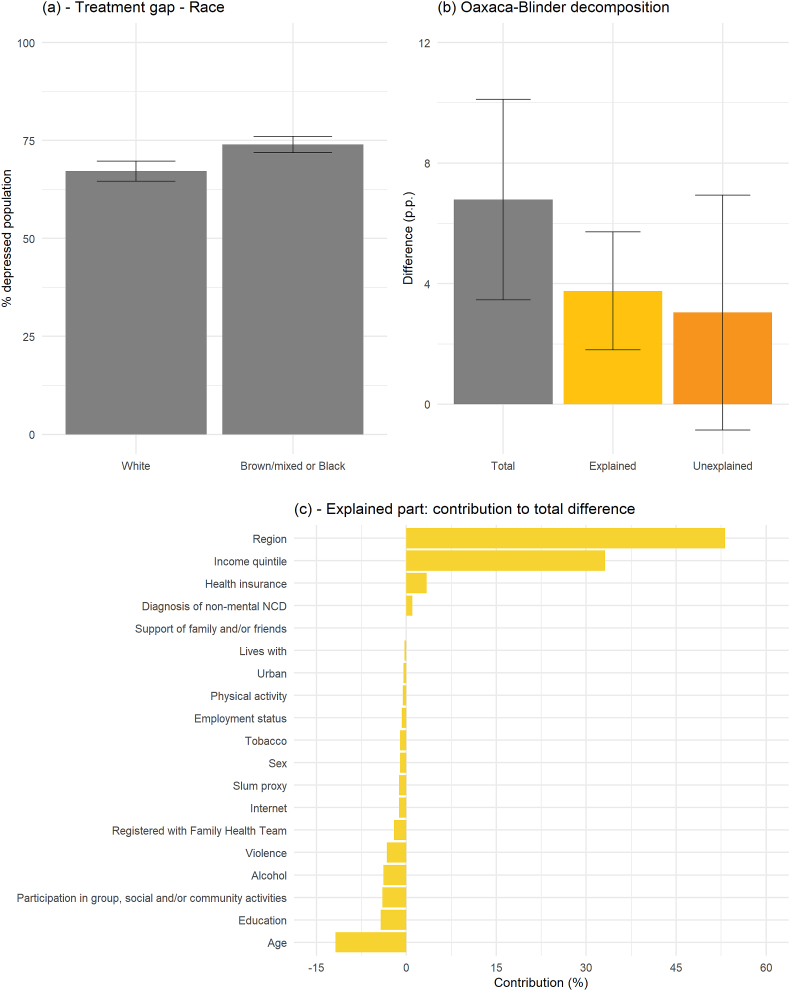
Oaxaca-Blinder decomposition for differences in the treatment gap for depression (PHQ9 ≥ 10) according to race Note: the figure shows the treatment gap for depression (PHQ9 ≥ 10) according to racial/ethnic self-identification for White and Brown/mixed or Black individuals (panel a). Additionally, it shows results from an Oaxaca-Blinder decomposition of the differences in the treatment gap for depression between the two groups (panel b). Error bars display 95% confidence intervals. The percentual contribution of the explained part of each contributing factor to the total difference are displayed in panel c –for covariates with more than one category, the bars display the sum of the percentual contribution of all categories. Detailed results are shown in Table A6 in the Appendix. All reported data are weighted considering the sampling design. (For interpretation of the references to colour in this figure legend, the reader is referred to the Web version of this article.)

Examining factors explaining differences in the treatment gap between white individuals and brown/mixed or black individuals was carried out through Oaxaca-Blinder decomposition (Fig. 4; Table A6 in the Appendix). Differences in observable characteristics accounted for 55.4% of the total difference in the treatment gap between racial/ethnic groups. The most relevant driver of racial/ethnic differences in the treatment gap were region of residence, which explained 53.2% of observed differences between the two groups, and income quintile, which explained 33.1%. Notably, 44.6% of the difference in the treatment gap between white individuals and black and brown/mixed individuals was not explained by differences in observables. While differences in the treatment gap according to race/ethnicity are larger combining individuals with a PHQ-9 score ≥10 or currently receiving treatment, results from the decomposition analysis are similar and also identify region of residence as the largest contributing factor (Figure A8 in the Appendix).

Consistent with that, we observe that when excluding region dummies from the analysis, the share of explained variation in racial/ethnic inequality decreases (Figure A9 in the Appendix). While we cannot account for all possible non-observable confounding factors, we conjecture that differences in the availability of mental healthcare services across Brazilian regions is a significant factor underneath the role of region dummies in the analysis. Figure A10 in the Appendix shows the results from an exercise that follows the same analysis from Fig. 4, but replaces region of residence dummies by the number of mental health professionals (psychologists plus psychiatrists) per 100,000 residents and the Human Development Index in the state of residence. Results suggest that region of residence is relevant for differences in the treatment gap because of differences in availability of mental healthcare services (e.g., availability of health professionals) across regions rather than because of general living standards (e.g., as captured by the Human Development Index).

## 4 Discussion

The prevalence of depression in Brazil increased between 2013 and 2019, with 10.8% of adults depressed in 2019. Whilst there were modest declines in the treatment gap, over 70% depressed individuals in 2019 did not receive care. The fact that the prevalence of depression grew while the treatment gap decreased is not particularly surprising. Improvements in access to effective treatment by individuals affected with depression may have been limited while other drivers of mental health conditions may have remained a challenge. This may be the case of racial discrimination, lack of effective policies for mental health promotion and prevention, and other social and economic determinants of health. Socioeconomic inequalities in the prevalence of depression and racial/ethnic inequalities in the treatment gap in Brazil remain large. Employment, age, exposure to violence, and physical activity were important drivers of income inequalities in depression, whilst region and income were important contributing factors for racial/ethnic inequalities in the treatment gap.

The findings on the prevalence of depression and the treatment gap are in line with studies showing that mental healthcare in Brazil remains a long-lasting challenge (Faisal-Cury et al., 2022; Lopes et al., 2016). Analyses from Argentina, Canada, Chile, Colombia, Guatemala, Mexico, Peru, and the United States showed that the mean treatment gap for any mental disorder was around 71%, and no smaller than 58% in any country, and that little progress has been made over time (Kohn et al., 2004, 2018). The findings from this study also showed that being a woman, having previous diagnosis of other NCDs and living in urban areas are positively associated with depression and that region of residence is a key determinant of the treatment gap, which is in line with previous results (Lopes et al., 2016).

Decomposition analysis showed that changes in observable characteristics could not account for the increase in the prevalence of depression and the decline in the treatment gap, which is surprising considering the large number of covariates included. Possible reasons could be economic shocks in Brazil between 2013 and 2019, general trends in improving mental healthcare access, or less stigma around mental health, however further studies are needed to identify specific driving factors.

There are notable findings around racial/ethnic groups. There were differences in the treatment gap across racial/ethnic groups with black or brown/mixed Brazilians most affected, but not in the prevalence of depression. In decomposition analyses, region was the most important determinant of the racial/ethnic inequalities in the treatment gap and this may be due to large regional differences in the racial/ethnic composition of populations, and the distribution of mental healthcare services and professionals. For example, in the South region –where 72.4% of individuals in our sample self-identified as white– there were 48 psychologists and psychiatrists per 100,000 residents in 2019. However, in the North and North-East regions –where 18.5% and 24.6% of individuals self-identified as white, respectively– there were only 17.6 and 24.8 psychologists and psychiatrists per 100,000 residents in 2019, respectively (Ministry of Health, 2021). Notably, a large share of inequalities in the treatment gap between white individuals and black and brown/mixed individuals was not explained by differences in observables, which could potentially be due to discrimination or difficulties in accessing treatment due to other non-observable characteristics.

Our results also show socioeconomic inequalities in depression prevalence and the treatment gap are large, but they are more pronounced for the former for our main definition of depression (PHQ-9 ≥ 10). One interpretation of these findings is that socio-economic and lifestyle factors drive inequalities in depression, but identifying and treating depression in individuals is a more cross-cutting challenge across all socioeconomic positions. The results from decomposition analysis support this as they show employment, age, exposure to violence and physical activity are the main contributing factors to income inequalities in depression. Factors that are more concentrated among higher-income individuals and positively associated with depression can act as levelers. This is the case of diagnosis of non-mental NCDs, which is higher among higher-income individuals, who are comparatively older and therefore have higher prevalence of NCDs. When individuals with high depressive symptoms (PHQ-9 ≥ 10) are grouped with individuals currently treated for depression, socioeconomic inequalities in the treatment gap are larger, and decomposition analysis suggests that region of residence is the largest contributing factor.

There are policy implications from this work. Policies aiming to reduce exposure of lower-income individuals to risk factors might have a positive impact on mental health and mental health inequalities. Brazil has the highest prevalence of insufficient physical activity in Latin America and the Caribbean (Guthold et al., 2018) and exposure to violence is highly prevalent and a major public health concern (Mascarenhas et al., 2021; Reichenheim et al., 2011). In Brazil, exposure to violence is intertwined with socioeconomic inequalities – it is higher in cities that are more segregated spatially (i.e., where higher-income and lower-income individuals tend to live in different areas) (Santos et al., 2021). It is also important to highlight that during our period of analysis homicides grew more in the poorer regions of the country (Machado et al., 2019). The socioeconomic gradient in physical inactivity, in turn, can be affected by social factors like urbanicity and transport infrastructure or conditions of daily living (Ball et al., 2015). Policies promoting physical activity and protecting against exposure to violence could have beneficial impacts not only on reducing mental health inequality but also on wider health and wellbeing improvements.

In societies where structural racism is prominent, formal and informal institutional arrangements shape the distribution of social determinants of health in prejudice of some racial/ethnic groups (Bailey et al., 2017). In Brazil, one way this manifests itself is in an unequal regional distribution of healthcare resources, in prejudice of regions with a higher share of black or brown/mixed individuals in the population. Addressing the regional unequal distribution of mental health services and professionals might be relevant for addressing racial/ethnic inequalities in the treatment gap. In the short term, a feasible alternative proven in other contexts (Baranov et al., 2020; Patel et al., 2018) could be to train community health workers to deliver psychosocial interventions. These professionals are largely available and geographically more evenly distributed in Brazil than other health professionals.

Key strengths of this study include the use of a large, recent, and nationally representative survey, and the use of the internationally-validated PHQ-9 screening tool for depression which is independent of medical diagnosis (Kroenke et al., 2001; Levis et al., 2019). Additionally, the novel use of decomposition methods allowed for identification of factors contributing to inequalities. However, there are limitations to the study. First, the nature of the study does not make it possible to make causal statements about the relationship between contributing factors and inequalities in depression and the treatment gap. Second, while the PHQ-9 is a widely used instrument and it has been shown to have high sensitivity and specificity in Brazil (Santos et al., 2013), it does not provide a clinical diagnosis of depression. Thirdly, the PNS only samples individuals in permanent households, with vulnerable homeless individuals excluded – potentially underestimating depression prevalence and associations identified. Finally, changes in the definition of variables and of response categories made it impossible to include exposure to violence as a covariate in the decomposition analysis of the evolution of the prevalence of depression. As it is a factor strongly correlated with the probabilities of being depressed, the large unexplained component in the analysis of trends might be related to its omission.

In spite of those limitations, this study provides a comprehensive picture of the challenges related to the growing prevalence of depression, the large size of the treatment gap and related socioeconomic and racial/ethnic inequalities. Results point to the necessity of further studies to understand the non-observable drivers behind large increases in the prevalence of depression. In relation to inequalities, the study points to the necessity of investing in policies to reduce the exposure of the poorer to risk factors, like physical inactivity and violence, of increasing the supply of mental healthcare in underserved regions, as well as of tackling barriers to access to healthcare stemming from discrimination.

## Ethical statement

All data used were collected by the Brazilian Institute of Geography and Statistics (IBGE) in partnership with the Brazilian Ministry of Health. All data used are anonymized, non-identifiable and publicly available online and therefore its use is exempt from ethical approval by an ethical review board.

## Author statement

**Matías Mrejen:** Conceptualization, Methodology, Data Curation, Formal Analysis, Writing - Original Draft.

**Thomas Hone:** Conceptualization, Writing - Review & Editing.

**Rudi Rocha:** Conceptualization, Methodology, Writing - Review & Editing.

## Declaration of competing interest

None.

## Data Availability

Data will be made available on request.
