# Supplementary material for: Socioeconomic and racial/ethnic inequalities in depression prevalence and the treatment gap in Brazil: A decomposition analysis
Source: SSM Popul Health. 2022 Oct 11;20:101266. doi: 10.1016/j.ssmph.2022.101266 (PMC9587003; doi:10.1016/j.ssmph.2022.101266)
Supplement: Multimedia component 1 [file mmc1.docx]

**Appendix**

**Table A1 – Summary statistics, subsample of depressed individuals (PHQ9 ≥ 10)**

| **Variable** | **2013** | | **2019** | |
| --- | --- | --- | --- | --- |
|  | Mean | 95%CI | Mean | 95%CI |
|  |  |  |  |  |
| **Depression (PHQ9 ≥ 10)** | 1.000 | 1.000 - 1.000 | 1.000 | 1.000 - 1.000 |
| **Treatment gap** | 0.761 | 0.740 - 0.781 | 0.712 | 0.695 - 0.728 |
|  |  |  |  |  |
| **Woman** | 0.722 | 0.701 - 0.743 | 0.737 | 0.722 - 0.752 |
| **Age** |  |  |  |  |
| 18-24 | 0.114 | 0.099 - 0.129 | 0.142 | 0.128 - 0.157 |
| 25-34 | 0.168 | 0.152 - 0.184 | 0.161 | 0.148 - 0.173 |
| 35-44 | 0.192 | 0.175 - 0.209 | 0.197 | 0.184 - 0.211 |
| 45-54 | 0.226 | 0.204 - 0.248 | 0.194 | 0.181 - 0.207 |
| 55-64 | 0.149 | 0.132 - 0.165 | 0.158 | 0.145 - 0.171 |
| 65 or older | 0.152 | 0.136 - 0.168 | 0.148 | 0.137 - 0.159 |
| **Race** |  |  |  |  |
| White | 0.452 | 0.429 - 0.475 | 0.423 | 0.404 - 0.441 |
| Black | 0.100 | 0.087 - 0.112 | 0.124 | 0.113 - 0.136 |
| Asian | 0.009 | 0.005 - 0.014 | 0.008 | 0.003 - 0.013 |
| Browns/Mixed | 0.435 | 0.412 - 0.459 | 0.439 | 0.421 - 0.457 |
| Indigenous | 0.004 | 0.002 - 0.006 | 0.006 | 0.004 - 0.008 |
| **Education** |  |  |  |  |
| None | 0.193 | 0.176 - 0.210 | 0.073 | 0.065 - 0.081 |
| Basic incomplete | 0.313 | 0.291 - 0.336 | 0.325 | 0.308 - 0.341 |
| Basic complete | 0.106 | 0.090 - 0.121 | 0.079 | 0.069 - 0.088 |
| Secondary incomplete | 0.047 | 0.038 - 0.056 | 0.075 | 0.064 - 0.085 |
| Secondary complete | 0.219 | 0.199 - 0.238 | 0.256 | 0.240 - 0.272 |
| Higher incomplete | 0.030 | 0.023 - 0.037 | 0.061 | 0.051 - 0.072 |
| Higher complete | 0.092 | 0.078 - 0.107 | 0.132 | 0.118 - 0.145 |
| **Urban** | 0.888 | 0.874 - 0.901 | 0.902 | 0.893 - 0.911 |
| **Region** |  |  |  |  |
| North | 0.058 | 0.051 - 0.065 | 0.060 | 0.055 - 0.065 |
| North-East | 0.268 | 0.248 - 0.288 | 0.261 | 0.248 - 0.274 |
| South-East | 0.427 | 0.401 - 0.453 | 0.461 | 0.443 - 0.480 |
| South | 0.170 | 0.152 - 0.187 | 0.139 | 0.127 - 0.150 |
| Center-West | 0.077 | 0.068 - 0.086 | 0.079 | 0.073 - 0.086 |
| **Slum proxy** | 0.205 | 0.185 - 0.224 | 0.159 | 0.146 - 0.171 |
| **Internet** | 0.412 | 0.388 - 0.435 | 0.848 | 0.837 - 0.859 |
| **Employment status** |  |  |  |  |
| Inactive | 0.487 | 0.464 - 0.511 | 0.429 | 0.412 - 0.446 |
| Unemployed | 0.038 | 0.029 - 0.047 | 0.074 | 0.065 - 0.084 |
| Employed | 0.474 | 0.451 - 0.498 | 0.497 | 0.480 - 0.514 |
| **Log family income per capita (R$ 2019)** | 6.645 | 6.591 - 6.698 | 6.667 | 6.623 - 6.711 |
| **Support of family and/or friends** | 0.893 | 0.877 - 0.909 | 0.968 | 0.963 - 0.974 |
| **Lives with** |  |  |  |  |
| Alone | 0.073 | 0.065 - 0.082 | 0.082 | 0.075 - 0.088 |
| Partner | 0.598 | 0.576 - 0.620 | 0.540 | 0.521 - 0.558 |
| Other person | 0.329 | 0.308 - 0.350 | 0.379 | 0.361 - 0.397 |
| **Participation in group, social and/or community activities** |  |  |  |  |
| Never | 0.237 | 0.217 - 0.258 | 0.196 | 0.181 - 0.210 |
| Less than monthly | 0.205 | 0.186 - 0.224 | 0.209 | 0.194 - 0.223 |
| At least once per month | 0.558 | 0.534 - 0.582 | 0.596 | 0.578 - 0.613 |
| **Health insurance** | 0.248 | 0.226 - 0.270 | 0.268 | 0.249 - 0.286 |
| **Registered with Family Health Team** |  |  |  |  |
| No / Does not know | 0.417 | 0.390 - 0.444 | 0.362 | 0.340 - 0.383 |
| Yes, and no home visits in last 12 months | 0.105 | 0.091 - 0.119 | 0.171 | 0.157 - 0.184 |
| Yes, and at least one home visit in last 12 months | 0.478 | 0.451 - 0.505 | 0.467 | 0.446 - 0.489 |
| **Diagnosis of non-mental NCD** | 0.756 | 0.736 - 0.775 | 0.752 | 0.736 - 0.769 |
| **Tobacco** | 0.202 | 0.183 - 0.220 | 0.171 | 0.157 - 0.185 |
| **Physical activity** |  |  |  |  |
| No | 0.790 | 0.771 - 0.809 | 0.683 | 0.666 - 0.701 |
| Yes, less than weekly | 0.011 | 0.005 - 0.017 | 0.017 | 0.012 - 0.022 |
| Yes, once or twice a week | 0.070 | 0.059 - 0.080 | 0.101 | 0.088 - 0.114 |
| Yes, three or more times per week | 0.129 | 0.113 - 0.145 | 0.199 | 0.184 - 0.214 |
| **Alcohol** |  |  |  |  |
| Never | 0.701 | 0.679 - 0.722 | 0.647 | 0.629 - 0.665 |
| Yes, less than weekly | 0.126 | 0.111 - 0.142 | 0.146 | 0.134 - 0.159 |
| Yes, once a week | 0.075 | 0.062 - 0.088 | 0.092 | 0.081 - 0.104 |
| Yes, twice or more per week | 0.098 | 0.083 - 0.113 | 0.114 | 0.102 - 0.126 |

Note: the table shows means and 95% confidence intervals for all variables used in the analysis for the subsample of individuals with depression. All reported data are weighted considering the sampling design.

**Figure A1 – Prevalence of Depression (PHQ9 ≥ 10) or Currently Treated and Treatment Gap (PHQ9 ≥ 10 or Currently treated)**


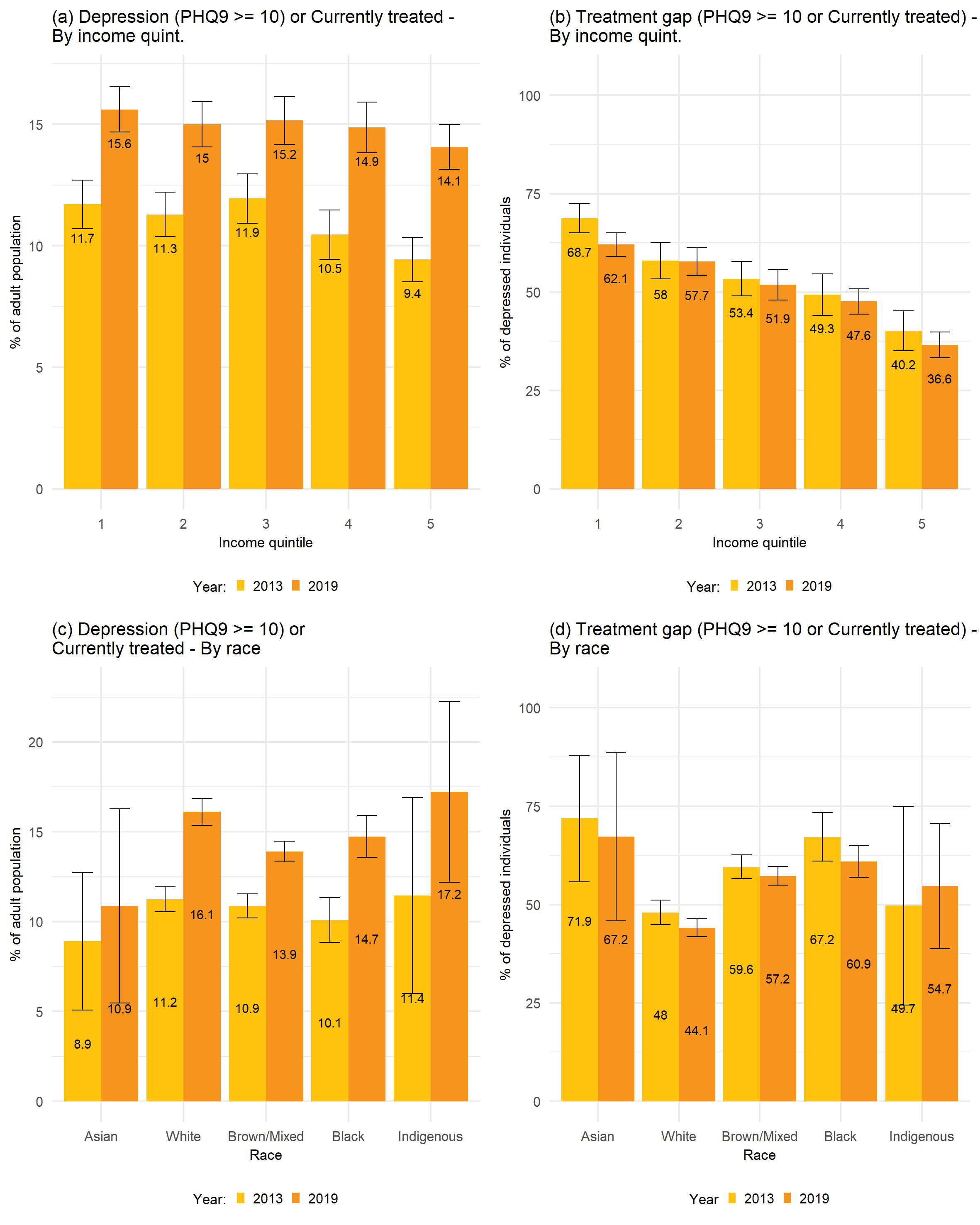


Note: the figure shows the evolution of the prevalence of an alternative measure grouping depression (PHQ9 ≥ 10) and individuals currently treated (panels a and c) and the treatment gap when considering that measure (panels b and d) in Brazil in 2013 and 2019 by income quintile and race/ethnicity. The error bars display the 95% confidence intervals. All reported data are weighted considering the sampling design.

**Table A2 – Linear probability model: depression (PHQ9 ≥ 10) and treatment gap for depression, 2013 and 2019**

|  | **Depression** | | | | **Treatment gap** | | | |
| --- | --- | --- | --- | --- | --- | --- | --- | --- |
|  | **2013** | | **2019** | | **2013** | | **2019** | |
|  | **Coef.** | **p.val** | **Coef.** | **p.val** | **Coef.** | **p.val** | **Coef.** | **p.val** |
| **Woman** | 0.044*** | 0.000 | 0.071*** | 0.000 | -0.020 | 0.424 | -0.062*** | 0.001 |
| **Age** | *omitted: 18-24* | | | | | | | |
| 25-34 | 0.002 | 0.702 | -0.009 | 0.257 | -0.019 | 0.568 | -0.041 | 0.164 |
| 35-44 | 0.003 | 0.622 | -0.013* | 0.090 | -0.064* | 0.080 | -0.089*** | 0.003 |
| 45-54 | 0.007 | 0.309 | -0.020** | 0.015 | -0.057 | 0.122 | -0.113*** | 0.000 |
| 55-64 | -0.024*** | 0.001 | -0.047*** | 0.000 | -0.069 | 0.108 | -0.113*** | 0.003 |
| 65 or older | -0.035*** | 0.000 | -0.075*** | 0.000 | 0.069 | 0.132 | 0.029 | 0.492 |
| **Race** | *omitted: White* | | | | | | | |
| Black | 0.004 | 0.558 | 0.004 | 0.500 | 0.003 | 0.925 | 0.052** | 0.039 |
| Asian | 0.008 | 0.643 | -0.019 | 0.486 | 0.051 | 0.469 | 0.102 | 0.294 |
| Browns/Mixed | 0.004 | 0.403 | -0.001 | 0.839 | 0.012 | 0.597 | 0.020 | 0.293 |
| Indigenous | -0.009 | 0.664 | 0.017 | 0.416 | -0.061 | 0.676 | 0.050 | 0.533 |
| **Education** | *omitted: None* | | | | | | | |
| Basic incomplete | -0.010 | 0.106 | -0.009 | 0.244 | -0.089*** | 0.001 | -0.030 | 0.332 |
| Basic complete | -0.016* | 0.055 | -0.018* | 0.066 | -0.077** | 0.048 | -0.047 | 0.267 |
| Secondary incomplete | -0.023*** | 0.006 | -0.006 | 0.615 | -0.082* | 0.082 | -0.094** | 0.041 |
| Secondary complete | -0.028*** | 0.000 | -0.028*** | 0.001 | -0.071** | 0.041 | -0.044 | 0.244 |
| Higher incomplete | -0.031*** | 0.000 | 0.008 | 0.543 | -0.124** | 0.038 | -0.040 | 0.441 |
| Higher complete | -0.023*** | 0.006 | -0.019** | 0.046 | -0.098** | 0.038 | -0.059 | 0.170 |
| **Urban** | 0.035*** | 0.000 | 0.039*** | 0.000 | -0.027 | 0.331 | 0.031 | 0.189 |
| **Region** | *omitted: North* | | | | | | | |
| North-East | 0.014*** | 0.004 | 0.017*** | 0.000 | -0.056*** | 0.009 | -0.061*** | 0.001 |
| South-East | 0.016*** | 0.005 | 0.025*** | 0.000 | -0.108*** | 0.000 | -0.148*** | 0.000 |
| South | 0.033*** | 0.000 | 0.020*** | 0.002 | -0.215*** | 0.000 | -0.204*** | 0.000 |
| Center-West | 0.025*** | 0.000 | 0.030*** | 0.000 | -0.059** | 0.028 | -0.106*** | 0.000 |
| **Slum proxy** | -0.007 | 0.169 | -0.004 | 0.453 | 0.003 | 0.896 | -0.013 | 0.502 |
| **Internet** | -0.010** | 0.033 | 0.011** | 0.034 | -0.052** | 0.047 | 0.018 | 0.423 |
| **Employment status** | *omitted: Inactive* | | | | | | | |
| Unemployed | -0.007 | 0.523 | 0.009 | 0.382 | 0.035 | 0.495 | 0.029 | 0.423 |
| Employed | -0.022*** | 0.000 | -0.025*** | 0.000 | 0.089*** | 0.000 | 0.069*** | 0.001 |
| **Log family income per capita (R$ 2019)** | -0.006*** | 0.001 | -0.008*** | 0.000 | -0.023*** | 0.000 | -0.022*** | 0.008 |
| **Support of family and/or friends** | -0.047*** | 0.000 | -0.056*** | 0.000 | -0.035 | 0.285 | -0.002 | 0.969 |
| **Lives with** | *omitted: Alone* | | | | | | | |
| Partner | -0.007 | 0.182 | -0.019*** | 0.000 | 0.048 | 0.102 | 0.023 | 0.297 |
| Other person | 0.001 | 0.815 | 0.003 | 0.568 | 0.044 | 0.149 | -0.011 | 0.644 |
| **Participation in group, social and/or community activities** | *omitted: Never* | | | | | | | |
| Less than monthly | -0.002 | 0.762 | -0.001 | 0.847 | -0.029 | 0.340 | 0.001 | 0.979 |
| At least once per month | -0.002 | 0.721 | -0.015*** | 0.008 | -0.015 | 0.523 | -0.056** | 0.012 |
| **Health insurance** | -0.006 | 0.230 | -0.002 | 0.698 | 0.004 | 0.884 | -0.051** | 0.027 |
| **Registered with Family Health Team** | *omitted: No / Does not know* | | | | | | | |
| Yes, and no home visits in last 12 months | 0.008 | 0.170 | 0.017*** | 0.002 | -0.026 | 0.477 | 0.015 | 0.522 |
| Yes, and at least one home visit in last 12 months | -0.001 | 0.903 | -0.001 | 0.887 | -0.016 | 0.465 | -0.015 | 0.427 |
| **Diagnosis of non-mental NCD** | 0.079*** | 0.000 | 0.096*** | 0.000 | -0.075*** | 0.001 | -0.104*** | 0.000 |
| **Tobacco** | 0.033*** | 0.000 | 0.045*** | 0.000 | -0.017 | 0.527 | -0.044* | 0.052 |
| **Physical activity** | *omitted: No* | | | | | | | |
| Yes, less than weekly | 0.006 | 0.765 | 0.009 | 0.597 | 0.104 | 0.131 | -0.003 | 0.957 |
| Yes, once or twice a week | -0.013*** | 0.006 | -0.027*** | 0.000 | 0.049 | 0.176 | -0.025 | 0.446 |
| Yes, three or more times per week | -0.021*** | 0.000 | -0.031*** | 0.000 | 0.023 | 0.459 | -0.007 | 0.744 |
| **Alcohol** | *omitted: Never* | | | | | | | |
| Yes, less than weekly | -0.015*** | 0.001 | -0.009* | 0.074 | 0.071** | 0.012 | 0.065*** | 0.006 |
| Yes, once a week | -0.013** | 0.016 | -0.013** | 0.026 | 0.123*** | 0.000 | 0.108*** | 0.000 |
| Yes, twice or more per week | -0.010* | 0.075 | -0.009 | 0.105 | 0.104*** | 0.003 | 0.104*** | 0.000 |
| **Constant** | 0.107*** | 0.000 | 0.151*** | 0.000 | 1.160*** | 0.000 | 1.132*** | 0.000 |
|  |  |  |  |  |  |  |  |  |
| **Observations** | 60,188 |  | 88,500 |  | 5,049 |  | 9,251 |  |
| **R-squared** | 0.049 |  | 0.058 |  | 0.091 |  | 0.079 |  |

Note: the table shows detailed results from four independent linear probability models linking covariates with the probability of being depressed and of falling in the treatment gap —conditional on being depressed— in 2013 and in 2019. All reported data are weighted considering the sampling design. * p-value < 0.1, ** p-value < 0.05, *** p-value < 0.01.

**Table A3 – Predicted probabilities: depression (PHQ9 ≥ 10) and treatment gap for depression, 2013 and 2019**

|  | **Depression** | | | | | | **Treatment gap** | | | | | |
| --- | --- | --- | --- | --- | --- | --- | --- | --- | --- | --- | --- | --- |
|  | **2013** | | | **2019** | | | **2013** | | | **2019** | | |
|  | **Pred. Prob.** | **95%CI** | **Pred. Prob.** | | **95%CI** | **Pred. Prob.** | | **95%CI** | **Pred. Prob.** | | **95%CI** |  |
| **Sex** |  |  |  | |  |  | |  |  | |  |  |
| Men | 0.055 | 0.050 - 0.060 | 0.071 | | 0.066 - 0.076 | 0.787 | | 0.756 - 0.819 | 0.771 | | 0.746 - 0.796 |  |
| Women | 0.099 | 0.094 - 0.105 | 0.142 | | 0.136 - 0.147 | 0.782 | | 0.760 - 0.803 | 0.702 | | 0.685 - 0.720 |  |
| **Age** |  |  |  | |  |  | |  |  | |  |  |
| 18-24 | 0.084 | 0.075 - 0.093 | 0.134 | | 0.121 - 0.148 | 0.780 | | 0.730 - 0.831 | 0.793 | | 0.752 - 0.834 |  |
| 25-34 | 0.086 | 0.079 - 0.093 | 0.126 | | 0.116 - 0.135 | 0.806 | | 0.767 - 0.845 | 0.749 | | 0.710 - 0.787 |  |
| 35-44 | 0.087 | 0.078 - 0.095 | 0.121 | | 0.113 - 0.129 | 0.748 | | 0.702 - 0.793 | 0.710 | | 0.670 - 0.750 |  |
| 45-54 | 0.091 | 0.081 - 0.102 | 0.114 | | 0.106 - 0.123 | 0.750 | | 0.710 - 0.791 | 0.688 | | 0.653 - 0.723 |  |
| 55-64 | 0.060 | 0.050 - 0.071 | 0.087 | | 0.078 - 0.097 | 0.747 | | 0.696 - 0.799 | 0.677 | | 0.634 - 0.720 |  |
| 65 or older | 0.049 | 0.036 - 0.062 | 0.059 | | 0.048 - 0.070 | 0.902 | | 0.846 - 0.958 | 0.810 | | 0.768 - 0.853 |  |
| **Race** |  |  |  | |  |  | |  |  | |  |  |
| White | 0.077 | 0.071 - 0.082 | 0.108 | | 0.102 - 0.114 | 0.774 | | 0.745 - 0.802 | 0.714 | | 0.689 - 0.740 |  |
| Black | 0.080 | 0.069 - 0.092 | 0.112 | | 0.102 - 0.122 | 0.798 | | 0.753 - 0.844 | 0.782 | | 0.745 - 0.818 |  |
| Asian | 0.085 | 0.051 - 0.119 | 0.090 | | 0.038 - 0.142 | 0.856 | | 0.759 - 0.952 | 0.841 | | 0.699 - 0.984 |  |
| Browns/Mixed | 0.080 | 0.074 - 0.087 | 0.107 | | 0.102 - 0.113 | 0.791 | | 0.764 - 0.818 | 0.739 | | 0.716 - 0.762 |  |
| Indigenous | 0.068 | 0.030 - 0.106 | 0.125 | | 0.085 - 0.164 | 0.697 | | 0.602 - 0.792 | 0.731 | | 0.598 - 0.865 |  |
| **Education** |  |  |  | |  |  | |  |  | |  |  |
| None | 0.096 | 0.086 - 0.107 | 0.124 | | 0.109 - 0.138 | 0.850 | | 0.803 - 0.896 | 0.785 | | 0.737 - 0.834 |  |
| Basic incomplete | 0.086 | 0.077 - 0.095 | 0.115 | | 0.107 - 0.123 | 0.780 | | 0.748 - 0.813 | 0.747 | | 0.720 - 0.775 |  |
| Basic complete | 0.081 | 0.069 - 0.093 | 0.106 | | 0.093 - 0.119 | 0.808 | | 0.758 - 0.859 | 0.732 | | 0.665 - 0.799 |  |
| Secondary incomplete | 0.074 | 0.061 - 0.086 | 0.118 | | 0.101 - 0.134 | 0.747 | | 0.671 - 0.823 | 0.694 | | 0.636 - 0.752 |  |
| Secondary complete | 0.068 | 0.062 - 0.074 | 0.095 | | 0.089 - 0.102 | 0.772 | | 0.735 - 0.810 | 0.729 | | 0.698 - 0.759 |  |
| Higher incomplete | 0.065 | 0.053 - 0.078 | 0.132 | | 0.111 - 0.153 | 0.726 | | 0.641 - 0.812 | 0.697 | | 0.621 - 0.773 |  |
| Higher complete | 0.074 | 0.063 - 0.085 | 0.105 | | 0.094 - 0.116 | 0.704 | | 0.622 - 0.786 | 0.727 | | 0.681 - 0.772 |  |
| **Area** |  |  |  | |  |  | |  |  | |  |  |
| Rural | 0.048 | 0.040 - 0.057 | 0.075 | | 0.067 - 0.083 | 0.836 | | 0.797 - 0.876 | 0.712 | | 0.670 - 0.754 |  |
| Urban | 0.084 | 0.079 - 0.088 | 0.114 | | 0.109 - 0.118 | 0.777 | | 0.758 - 0.797 | 0.737 | | 0.721 - 0.753 |  |
| **Region** |  |  |  | |  |  | |  |  | |  |  |
| North | 0.061 | 0.054 - 0.069 | 0.088 | | 0.080 - 0.095 | 0.893 | | 0.861 - 0.925 | 0.843 | | 0.815 - 0.872 |  |
| North-East | 0.076 | 0.069 - 0.083 | 0.105 | | 0.099 - 0.111 | 0.826 | | 0.800 - 0.853 | 0.789 | | 0.767 - 0.811 |  |
| South-East | 0.077 | 0.070 - 0.084 | 0.113 | | 0.106 - 0.120 | 0.769 | | 0.736 - 0.802 | 0.703 | | 0.674 - 0.733 |  |
| South | 0.094 | 0.083 - 0.105 | 0.107 | | 0.098 - 0.116 | 0.685 | | 0.642 - 0.729 | 0.671 | | 0.634 - 0.708 |  |
| Center-West | 0.086 | 0.077 - 0.095 | 0.118 | | 0.109 - 0.127 | 0.814 | | 0.784 - 0.844 | 0.732 | | 0.698 - 0.767 |  |
| **Slum proxy** |  |  |  | |  |  | |  |  | |  |  |
| No | 0.080 | 0.076 - 0.084 | 0.109 | | 0.105 - 0.113 | 0.780 | | 0.760 - 0.800 | 0.735 | | 0.719 - 0.750 |  |
| Yes | 0.073 | 0.065 - 0.082 | 0.105 | | 0.097 - 0.114 | 0.802 | | 0.767 - 0.836 | 0.733 | | 0.698 - 0.768 |  |
| **Internet** |  |  |  | |  |  | |  |  | |  |  |
| No | 0.083 | 0.078 - 0.089 | 0.099 | | 0.090 - 0.108 | 0.794 | | 0.767 - 0.821 | 0.717 | | 0.681 - 0.754 |  |
| Yes | 0.074 | 0.068 - 0.080 | 0.110 | | 0.106 - 0.114 | 0.770 | | 0.738 - 0.801 | 0.738 | | 0.722 - 0.754 |  |
| **Employment status** |  |  |  | |  |  | |  |  | |  |  |
| Inactive | 0.092 | 0.084 - 0.100 | 0.123 | | 0.115 - 0.131 | 0.714 | | 0.683 - 0.745 | 0.692 | | 0.666 - 0.719 |  |
| Unemployed | 0.085 | 0.065 - 0.105 | 0.132 | | 0.114 - 0.151 | 0.833 | | 0.757 - 0.908 | 0.728 | | 0.671 - 0.784 |  |
| Employed | 0.071 | 0.066 - 0.075 | 0.098 | | 0.093 - 0.103 | 0.840 | | 0.815 - 0.864 | 0.769 | | 0.746 - 0.791 |  |
| **Log family income per capita (R$ 2019)** |  |  |  | |  |  | |  |  | |  |  |
| **Support of family and/or friends** |  |  |  | |  |  | |  |  | |  |  |
| No | 0.122 | 0.104 - 0.141 | 0.163 | | 0.135 - 0.191 | 0.809 | | 0.760 - 0.858 | 0.736 | | 0.660 - 0.811 |  |
| Yes | 0.076 | 0.072 - 0.080 | 0.107 | | 0.103 - 0.111 | 0.781 | | 0.762 - 0.800 | 0.734 | | 0.719 - 0.749 |  |
| **Lives with** |  |  |  | |  |  | |  |  | |  |  |
| Alone | 0.083 | 0.073 - 0.093 | 0.119 | | 0.110 - 0.128 | 0.759 | | 0.704 - 0.814 | 0.734 | | 0.695 - 0.772 |  |
| Partner | 0.075 | 0.071 - 0.080 | 0.100 | | 0.095 - 0.105 | 0.795 | | 0.772 - 0.817 | 0.745 | | 0.724 - 0.766 |  |
| Other person | 0.084 | 0.077 - 0.091 | 0.122 | | 0.115 - 0.130 | 0.772 | | 0.740 - 0.804 | 0.718 | | 0.693 - 0.743 |  |
| **Participation in group, social and/or community activities** |  |  |  | |  |  | |  |  | |  |  |
| Never | 0.080 | 0.072 - 0.088 | 0.118 | | 0.108 - 0.128 | 0.800 | | 0.767 - 0.833 | 0.771 | | 0.739 - 0.803 |  |
| Less than monthly | 0.078 | 0.070 - 0.086 | 0.117 | | 0.108 - 0.126 | 0.763 | | 0.720 - 0.806 | 0.768 | | 0.741 - 0.796 |  |
| At least once per month | 0.078 | 0.073 - 0.083 | 0.103 | | 0.099 - 0.108 | 0.785 | | 0.762 - 0.807 | 0.709 | | 0.687 - 0.731 |  |
| **Health insurance** |  |  |  | |  |  | |  |  | |  |  |
| No | 0.080 | 0.075 - 0.085 | 0.109 | | 0.104 - 0.114 | 0.782 | | 0.761 - 0.803 | 0.749 | | 0.731 - 0.767 |  |
| Yes | 0.075 | 0.067 - 0.082 | 0.107 | | 0.099 - 0.115 | 0.792 | | 0.752 - 0.831 | 0.693 | | 0.656 - 0.731 |  |
| **Registered with Family Health Team** |  |  |  | |  |  | |  |  | |  |  |
| No / Does not know | 0.078 | 0.072 - 0.084 | 0.106 | | 0.100 - 0.112 | 0.799 | | 0.772 - 0.827 | 0.736 | | 0.712 - 0.760 |  |
| Yes, and no home visits in last 12 months | 0.087 | 0.076 - 0.097 | 0.123 | | 0.114 - 0.132 | 0.748 | | 0.690 - 0.806 | 0.759 | | 0.726 - 0.793 |  |
| Yes, and at least one home visit in last 12 months | 0.078 | 0.072 - 0.084 | 0.105 | | 0.099 - 0.112 | 0.779 | | 0.754 - 0.803 | 0.725 | | 0.701 - 0.749 |  |
| **Diagnosis of non-mental NCD** |  |  |  | |  |  | |  |  | |  |  |
| No | 0.040 | 0.036 - 0.044 | 0.056 | | 0.052 - 0.061 | 0.838 | | 0.811 - 0.866 | 0.794 | | 0.769 - 0.818 |  |
| Yes | 0.119 | 0.113 - 0.126 | 0.152 | | 0.146 - 0.158 | 0.763 | | 0.741 - 0.785 | 0.714 | | 0.695 - 0.732 |  |
| **Tobacco** |  |  |  | |  |  | |  |  | |  |  |
| No | 0.074 | 0.070 - 0.078 | 0.103 | | 0.099 - 0.107 | 0.786 | | 0.764 - 0.807 | 0.744 | | 0.728 - 0.760 |  |
| Yes | 0.107 | 0.096 - 0.117 | 0.148 | | 0.136 - 0.160 | 0.779 | | 0.742 - 0.817 | 0.694 | | 0.656 - 0.732 |  |
| **Physical activity** |  |  |  | |  |  | |  |  | |  |  |
| No | 0.084 | 0.079 - 0.089 | 0.120 | | 0.115 - 0.125 | 0.776 | | 0.755 - 0.797 | 0.736 | | 0.717 - 0.755 |  |
| Yes, less than weekly | 0.090 | 0.050 - 0.130 | 0.129 | | 0.097 - 0.160 | 0.931 | | 0.843 - 1.018 | 0.598 | | 0.465 - 0.730 |  |
| Yes, once or twice a week | 0.071 | 0.063 - 0.079 | 0.093 | | 0.083 - 0.104 | 0.833 | | 0.781 - 0.886 | 0.758 | | 0.715 - 0.800 |  |
| Yes, three or more times per week | 0.063 | 0.056 - 0.071 | 0.089 | | 0.082 - 0.096 | 0.788 | | 0.737 - 0.840 | 0.732 | | 0.695 - 0.769 |  |
| **Alcohol** |  |  |  | |  |  | |  |  | |  |  |
| Never | 0.084 | 0.079 - 0.089 | 0.113 | | 0.107 - 0.118 | 0.746 | | 0.721 - 0.770 | 0.699 | | 0.676 - 0.721 |  |
| Yes, less than weekly | 0.069 | 0.061 - 0.076 | 0.103 | | 0.095 - 0.112 | 0.835 | | 0.793 - 0.876 | 0.765 | | 0.731 - 0.799 |  |
| Yes, once a week | 0.071 | 0.062 - 0.081 | 0.100 | | 0.090 - 0.110 | 0.881 | | 0.843 - 0.919 | 0.805 | | 0.765 - 0.845 |  |
| Yes, twice or more per week | 0.074 | 0.064 - 0.084 | 0.103 | | 0.095 - 0.112 | 0.865 | | 0.822 - 0.909 | 0.812 | | 0.769 - 0.856 |  |

Note: the table shows predicted probabilities for all categories of categorical variables using results from the four independent linear probability models described in table A2, keeping all other variables at their means. All reported data are weighted considering the sampling design.

**Figure A2 – Predicted probabilities of depression (PHQ9 ≥ 10) by income quintile and race/ethnicity (2019)**


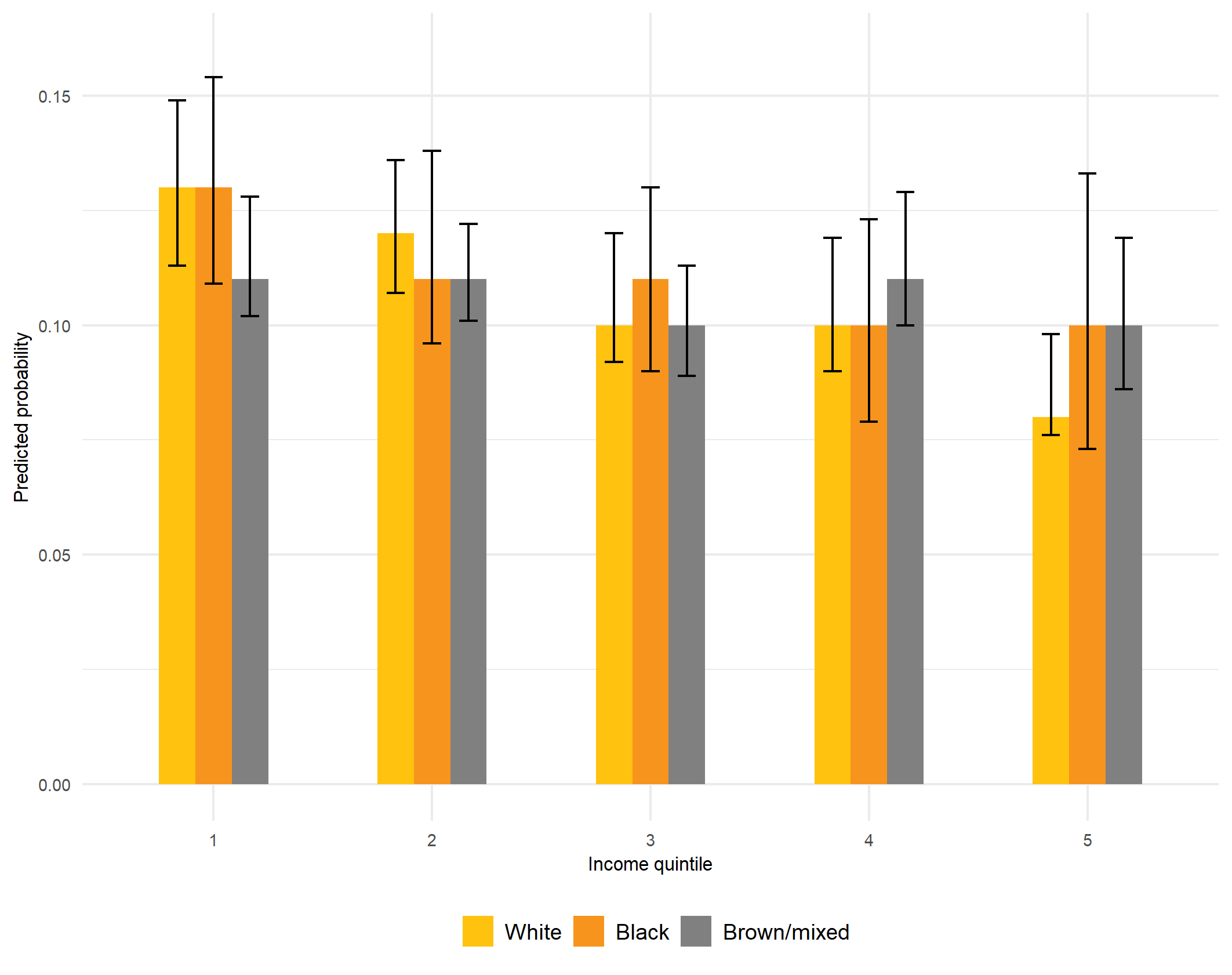


Note: the figure shows predicted probabilities of being depressed (PHQ9 ≥ 10) by income quintile and ethnicity/race for the three main racial/ethnic groups. Predicted probabilities were obtained from a model analogous to the one in Table A2, but the variable “Log family income per capita (R$ 2019)” was substituted by “Income quintile” and interacted with the variable “Race”. Results for Asian and Indigenous were omitted from the figure for clarity purposes. All reported data are weighted considering the sampling design.

**Figure A3 – Oaxaca-Blinder decomposition for the evolution of the prevalence of depression (PHQ9 ≥ 10) and the treatment gap for depression (2013 – 2019)**


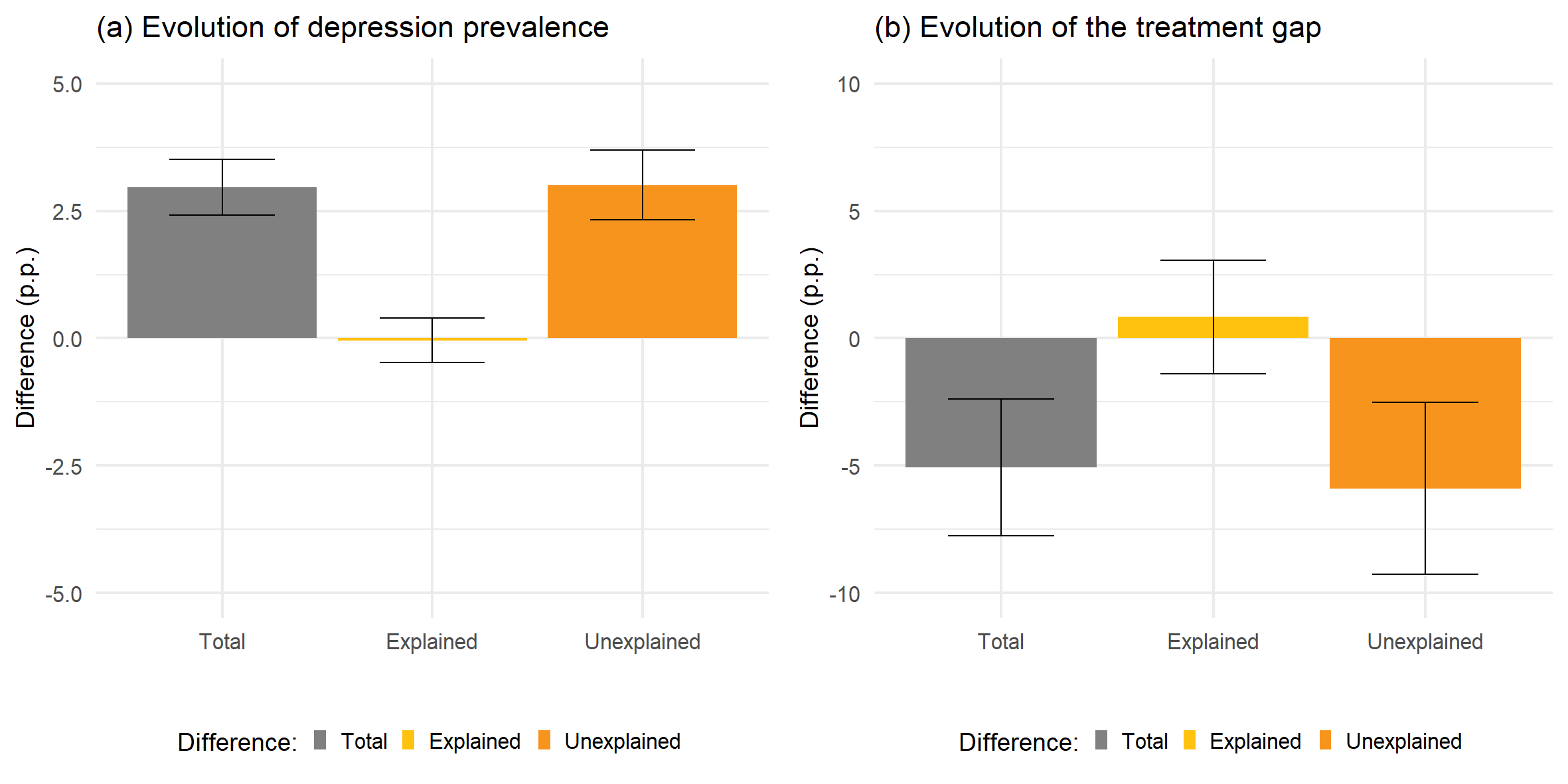


Note: the figure shows results from Oaxaca-Blinder decompositions of the evolution of the prevalence of depression (PHQ9 ≥ 10, panel a) and of the treatment gap for depression (panel b) between 2013 and 2019. Error bars display de 95% confidence interval. Full results from the decomposition are shown in Table A3 in the Appendix. For the analysis of the treatment gap, sampling strata without depressed individuals were dropped from regressions and therefore the mean is slightly different from the mean for the entire sample presented in Figure 1. All reported data are weighted considering the sampling design.

**Table A4 – Oaxaca-Blinder decomposition for the evolution of the prevalence of depression (PHQ9 ≥ 10) and the treatment gap for depression (2013 – 2019)**

|  | **Depression** | | **Treatment gap** | |
| --- | --- | --- | --- | --- |
|  |  |  |  |  |
| **2019** | 0.1083*** |  | 0.7117*** |  |
| **2013** | 0.0787*** |  | 0.7623*** |  |
|  |  |  |  |  |
| **Aggregate decomposition** | **Estimate** | **%** | **Estimate** | % |
| **Difference** | 0.0296*** |  | -0.0506*** |  |
| **Explained** | -0.0004 | -1.35% | 0.0084 | -16.60% |
| **Unexplained** | 0.0301*** | 101.69% | -0.0590*** | 116.60% |
|  |  |  |  |  |
| **Detailed decomposition** | **Explained** | | | |
|  | **Estimate** | **%** | **Estimate** | % |
| **Sex** | 0.0002*** | 0.68% | -0.0010 | 1.98% |
| **Age** | -0.0026*** | -8.78% | 0.0023 | -4.55% |
| **Race** | 0.0001 | 0.34% | 0.0012 | -2.37% |
| **Education** | -0.0010 | -3.38% | -0.0070 | 13.83% |
| **Urban** | 0.0000 | 0.00% | 0.0005 | -0.99% |
| **Region** | -0.0001*** | -0.34% | 0.0008 | -1.58% |
| **Slum proxy** | 0.0001 | 0.34% | 0.0006 | -1.19% |
| **Internet** | 0.0038** | 12.84% | 0.0078 | -15.42% |
| **Employment status** | 0.0002 | 0.68% | 0.0027 | -5.34% |
| **Log family income per capita (R$ 2019)** | 0.0002 | 0.68% | -0.0007 | 1.38% |
| **Support of family and/or friends** | -0.0026*** | -8.78% | -0.0001 | 0.20% |
| **Lives with** | -0.0001 | -0.34% | -0.0019* | 3.75% |
| **Participation in group, social and/or community activities** | -0.0010*** | -3.38% | -0.0021* | 4.15% |
| **Health insurance** | 0.0000 | 0.00% | -0.0011 | 2.17% |
| **Registered with Family Health Team** | 0.0008** | 2.70% | 0.0012 | -2.37% |
| **Diagnosis of non-mental NCD** | 0.0055*** | 18.58% | 0.0004 | -0.79% |
| **Tobacco** | -0.0009*** | -3.04% | 0.0013 | -2.57% |
| **Physical activity** | -0.0029*** | -9.80% | -0.0013 | 2.57% |
| **Alcohol** | -0.0002* | -0.68% | 0.0049*** | -9.68% |

Note: the table shows full results from Oaxaca-Blinder decompositions of the evolution of the prevalence of depression (PHQ9 ≥ 10) and the treatment gap for depression between 2013 and 2019. Results for categorical variables are the sum of the results of each category. For the analysis of the treatment gap, sampling strata without depressed individuals were dropped from regressions and therefore the mean is slightly different from the mean for the entire sample presented in Figure 1. All reported data are weighted considering the sampling design. * p-value < 0.1, ** p-value < 0.05, *** p-value < 0.01.

**Table A5 – Decomposition of the Concentration Index for economic inequalities in depression prevalence (PHQ9 ≥ 10), 2019**

| **Variable** | **Regression coefficient** | **Mean** | **C** | **Contribution** | **Contribution %** |
| --- | --- | --- | --- | --- | --- |
| **Woman** | 0.0660*** | 0.5316 | -0.0228 | -0.0032 | 8.62% |
| **Age** |  |  |  |  |  |
| 25-34 | 0.0021 | 0.1810 | -0.0800 | -0.0001 | 0.33% |
| 35-44 | 0.0012 | 0.2024 | -0.0652 | -0.0001 | 0.17% |
| 45-54 | 0.0018 | 0.1785 | 0.0348 | 0.0000 | -0.12% |
| 55-64 | -0.0208** | 0.1505 | 0.1139 | -0.0014 | 3.84% |
| 65 or older | -0.0448*** | 0.1490 | 0.1929 | -0.0052 | 13.88% |
| **Race** |  |  |  |  |  |
| Black | 0.0002 | 0.1147 | -0.1461 | 0.0000 | 0.04% |
| Asian | -0.0180 | 0.0092 | 0.1877 | -0.0001 | 0.34% |
| Browns/Mixed | -0.0039 | 0.4380 | -0.1603 | 0.0011 | -2.95% |
| Indigenous | 0.0079 | 0.0054 | -0.2179 | 0.0000 | 0.10% |
| **Education** |  |  |  |  |  |
| Basic incomplete | -0.0090 | 0.2866 | -0.1956 | 0.0020 | -5.44% |
| Basic complete | -0.0159* | 0.0776 | -0.1150 | 0.0006 | -1.53% |
| Secondary incomplete | -0.0082 | 0.0672 | -0.2292 | 0.0005 | -1.36% |
| Secondary complete | -0.0256*** | 0.2981 | -0.0100 | 0.0003 | -0.82% |
| Higher incomplete | 0.0063 | 0.0512 | 0.2495 | 0.0003 | -0.87% |
| Higher complete | -0.0180* | 0.1583 | 0.5473 | -0.0062 | 16.81% |
| **Urban** | 0.0325*** | 0.8618 | 0.0568 | 0.0064 | -17.15% |
| **Region** |  |  |  |  |  |
| North-East | 0.0149*** | 0.2645 | -0.2841 | -0.0045 | 12.07% |
| South-East | 0.0241*** | 0.4344 | 0.1341 | 0.0056 | -15.14% |
| South | 0.0198*** | 0.1468 | 0.1978 | 0.0023 | -6.20% |
| Center-West | 0.0305*** | 0.0757 | 0.1002 | 0.0009 | -2.49% |
| **Slum proxy** | -0.0038 | 0.1514 | -0.1982 | 0.0005 | -1.23% |
| **Internet** | 0.0080 | 0.8459 | 0.0476 | 0.0013 | -3.47% |
| **Employment status** |  |  |  |  |  |
| Unemployed | 0.0057 | 0.0527 | -0.4217 | -0.0005 | 1.37% |
| Employed | -0.0312*** | 0.6125 | 0.0866 | -0.0066 | 17.84% |
| **Income quintile** |  |  |  |  |  |
| Q2 | -0.0033 | 0.2032 | -0.3956 | 0.0011 | -2.86% |
| Q3 | -0.0132** | 0.1963 | 0.0039 | 0.0000 | 0.11% |
| Q4 | -0.0088 | 0.2108 | 0.4109 | -0.0030 | 8.22% |
| Q5 | -0.0242*** | 0.1891 | 0.8109 | -0.0148 | 40.01% |
| **Support of family and/or friends** | -0.0455*** | 0.9815 | 0.0043 | -0.0008 | 2.07% |
| **Lives with** |  |  |  |  |  |
| Partner | -0.0142*** | 0.6139 | 0.0011 | 0.0000 | 0.10% |
| Other person | 0.0033 | 0.3112 | -0.0752 | -0.0003 | 0.83% |
| **Participation in group, social and/or community activities** |  |  |  |  |  |
| Less than monthly | -0.0074 | 0.1886 | -0.0588 | 0.0003 | -0.88% |
| At least once per month | -0.0223*** | 0.6442 | 0.0436 | -0.0025 | 6.75% |
| **Health insurance** | 0.0003 | 0.2964 | 0.4177 | 0.0001 | -0.40% |
| **Registered with Family Health Team** |  |  |  |  |  |
| Yes, and no home visits in last 12 months | 0.0153*** | 0.1442 | 0.0198 | 0.0002 | -0.47% |
| Yes, and at least one home visit in last 12 months | 0.0012 | 0.4711 | -0.1486 | -0.0003 | 0.91% |
| **Violence** | 0.1479*** | 0.1827 | -0.0524 | -0.0057 | 15.27% |
| **Diagnosis of non-mental NCD** | 0.0833*** | 0.5442 | 0.0451 | 0.0082 | -22.04% |
| **Tobacco** | 0.0374*** | 0.1259 | -0.0952 | -0.0018 | 4.83% |
| **Physical activity** |  |  |  |  |  |
| Yes, less than weekly | 0.0004 | 0.0155 | 0.1336 | 0.0000 | -0.01% |
| Yes, once or twice a week | -0.0274*** | 0.1467 | 0.0795 | -0.0013 | 3.45% |
| Yes, three or more times per week | -0.0297*** | 0.2582 | 0.1700 | -0.0052 | 14.06% |
| **Alcohol** |  |  |  |  |  |
| Yes, less than weekly | -0.0113** | 0.1578 | 0.0157 | -0.0001 | 0.30% |
| Yes, once a week | -0.0154*** | 0.1179 | 0.1161 | -0.0008 | 2.27% |
| Yes, twice or more per week | -0.0157*** | 0.1460 | 0.1766 | -0.0016 | 4.36% |
|  |  |  |  |  |  |
| **Explained** |  |  |  | -0.0347 | 93.51% |
| **Residual** |  |  |  | -0.0024 | 6.49% |
| **E** |  |  | -0.0371 |  |  |

Note: the table shows the detailed decomposition of the Erreygers-corrected concentration index in 2019. The first three columns show: the coefficients of the association between all covariates and the probabilities of being depressed (PHQ9 ≥ 10) obtained from a linear probability model, the mean, and the concentration index of each covariate. The contribution of each factor is equal to four times the product of the regression coefficient, the mean and the concentration index. All reported data are weighted considering the sampling design. * p-value < 0.1, ** p-value < 0.05, *** p-value < 0.01.

**Figure A4 – Decomposition of the Erreygers-corrected concentration index for Depression (PHQ9 ≥ 10) (excl. exposure to violence)**


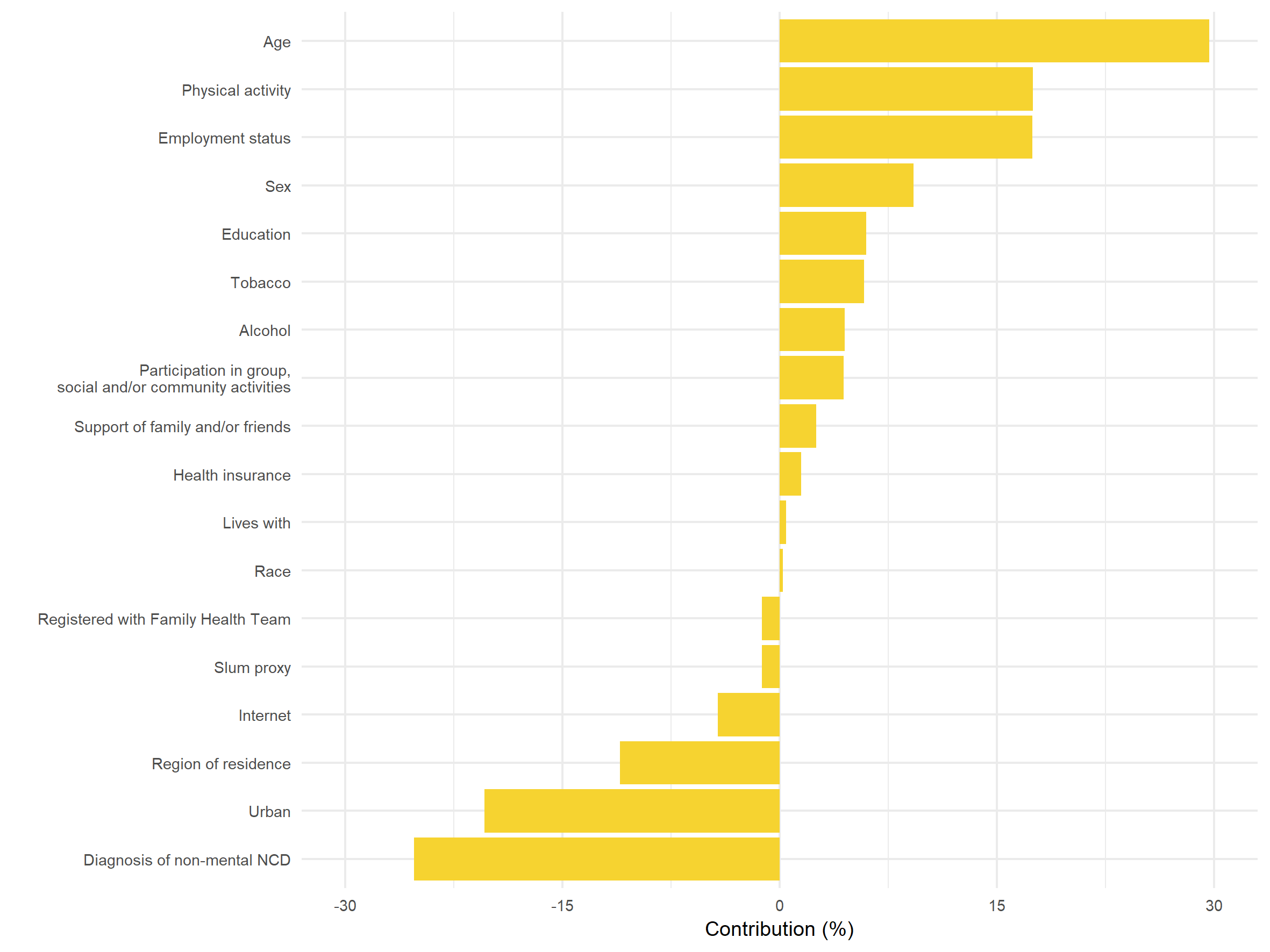


Note: the figure shows the results from the decomposition of the Erreygers-corrected concentration index for depression (PHQ9 ≥ 10) in 2019. Variables included were the same as in Figure 3 and Table A5, but excluding exposure to violence. All reported data are weighted considering the sampling design.


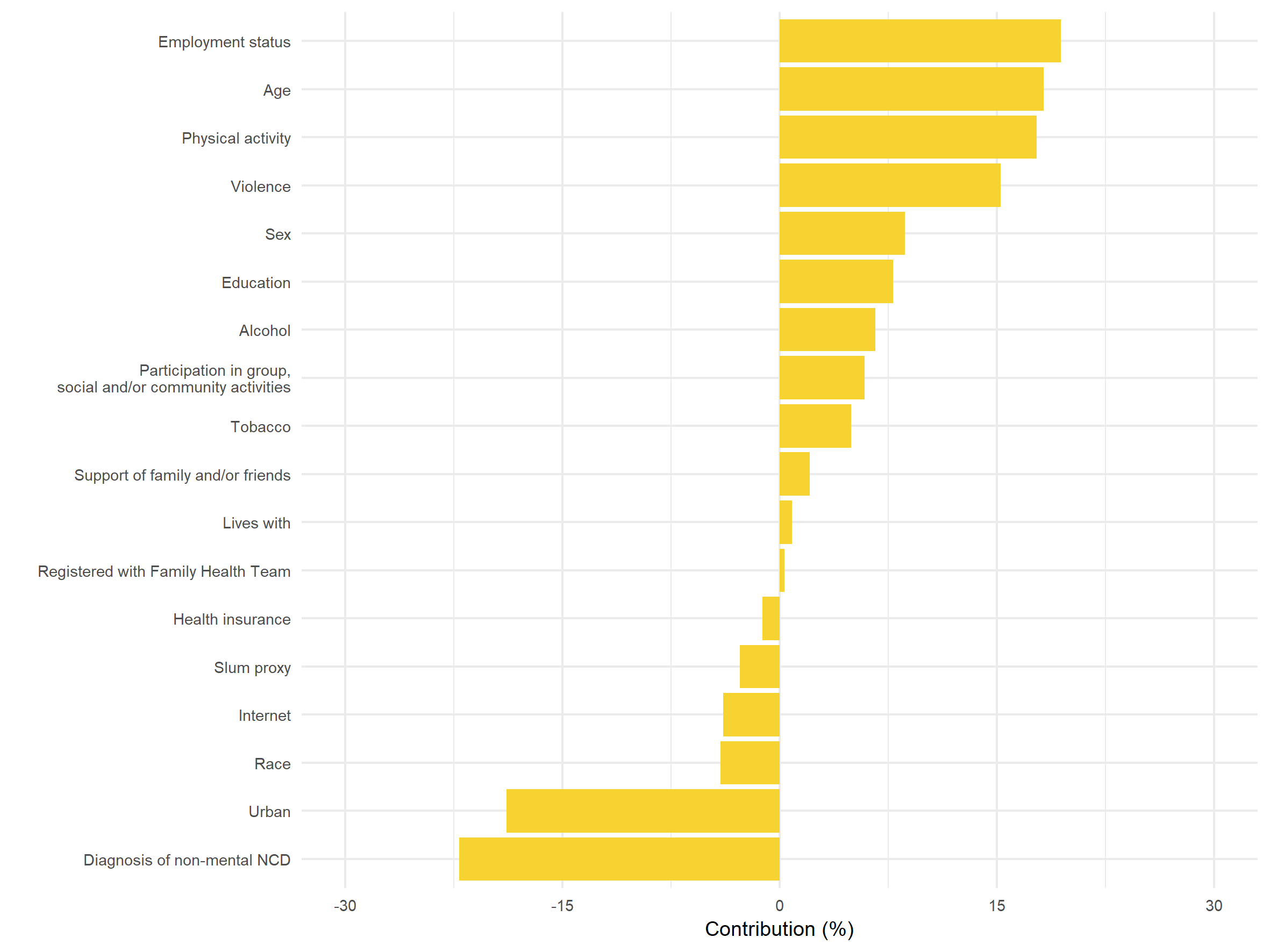
**Figure A5 – Decomposition of the Erreygers-corrected concentration index for Depression (PHQ9 ≥ 10) (excl. region)**

Note: the figure shows the results from the decomposition of the Erreygers-corrected concentration index for depression (PHQ9 ≥ 10) in 2019. Variables included were the same as in Figure 3 and Table A5, but excluding region of residence. All reported data are weighted considering the sampling design.


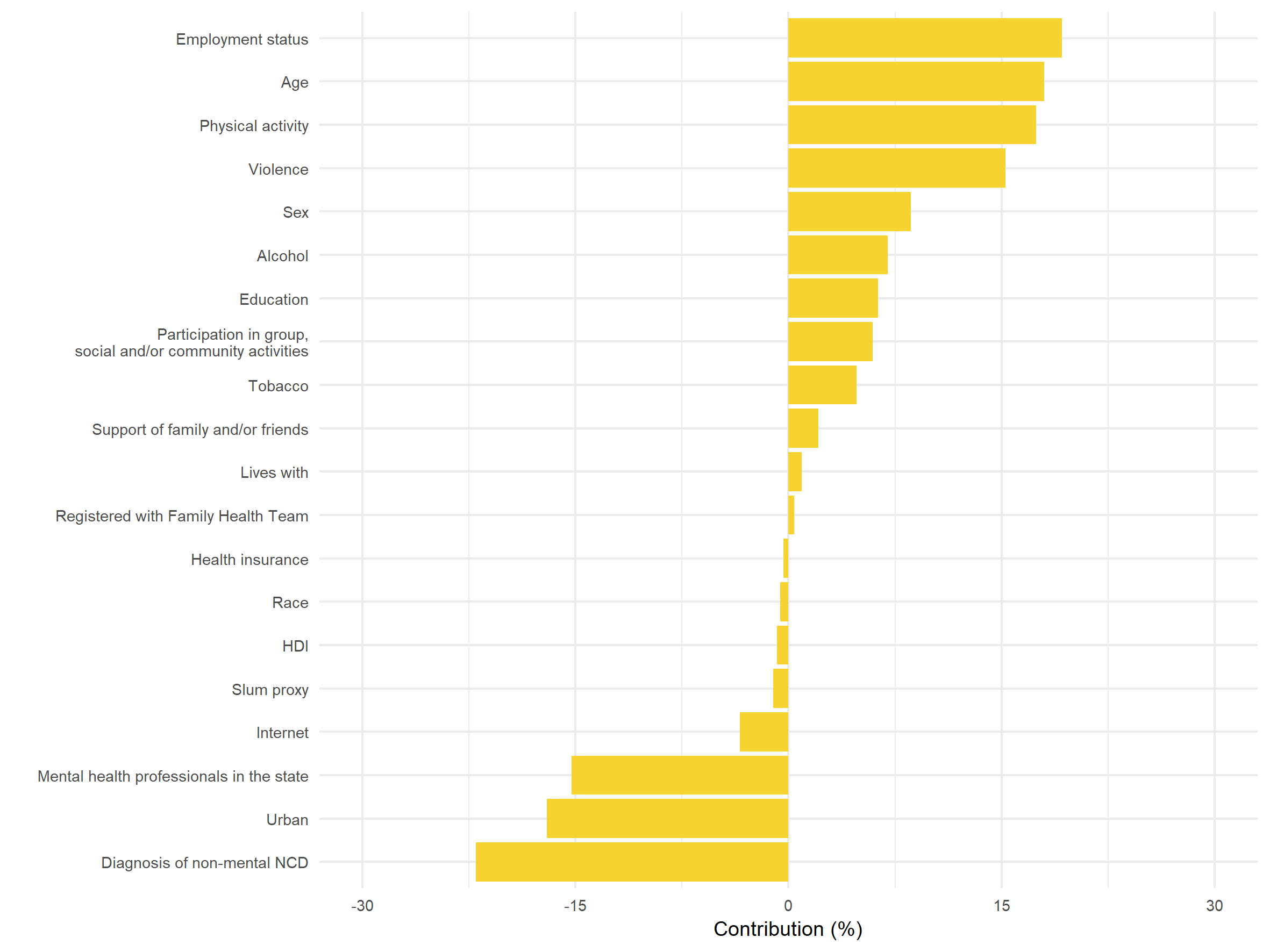
**Figure A6 – Decomposition of the Erreygers-corrected concentration index for Depression (PHQ9 ≥ 10) (incl. rate of mental health professionals and HDI)**

Note: the figure shows the results from the decomposition of the Erreygers-corrected concentration index for depression (PHQ9 ≥ 10) in 2019. Variables included were the same as in Figure 3 and Table A5, but substituting region of residence for the rate of mental health professionals and HDI in the state of residence in the analysis. All reported data are weighted considering the sampling design.

**Figure A7 – Concentration Curve and decomposition of the Erreygers-corrected concentration index for the treatment gap (PHQ9 ≥ 10 or currently treated)**


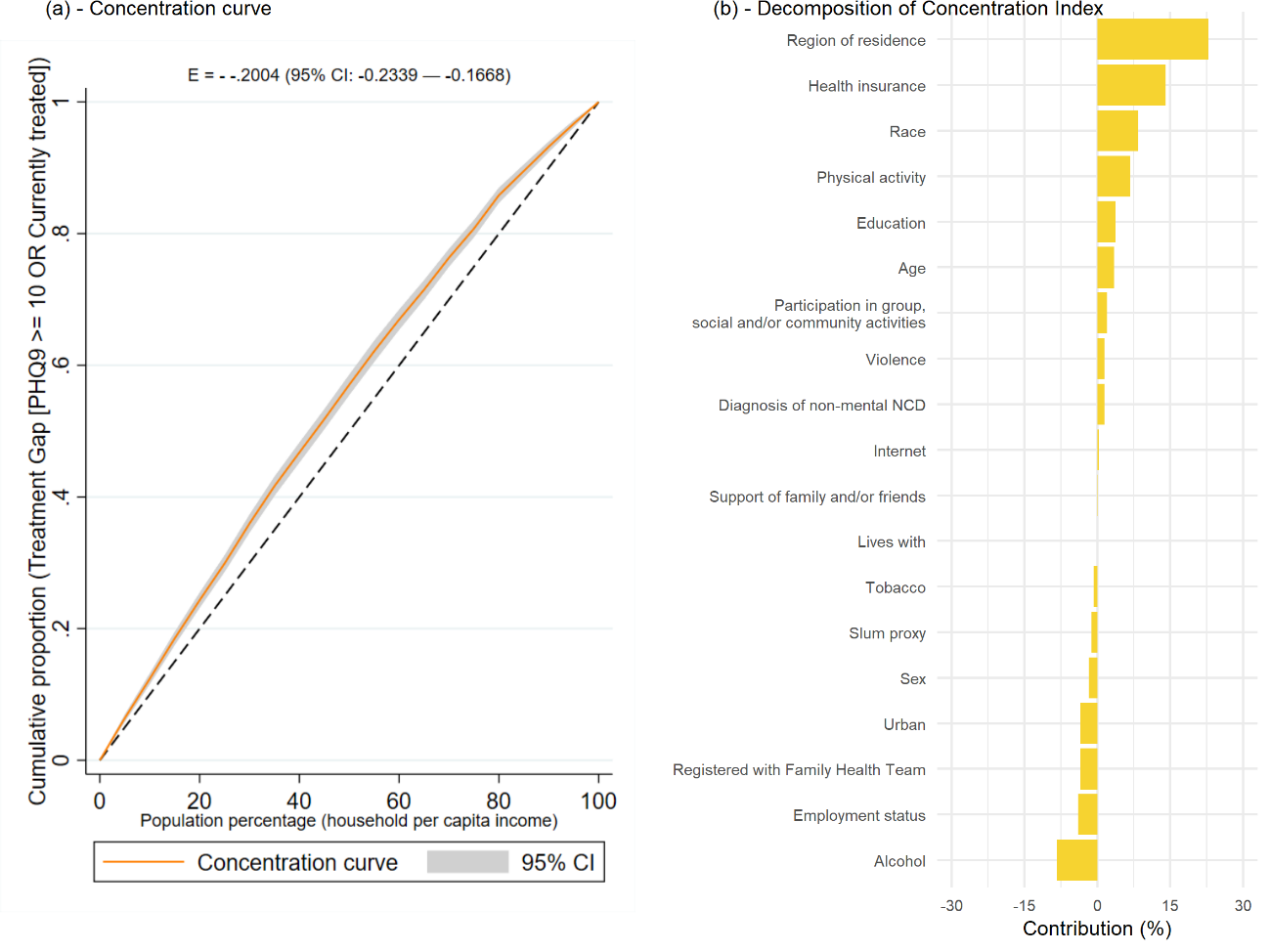


Note: the figure shows the concentration curve and the Erreygers-corrected concentration index for the treatment gap in 2019 (panel a, grouping individuals with depression (PHQ9 ≥ 10) or currently treated and results from the decomposition of the concentration index (panel b). A variable indicating income quintile according to per capita household income was included in the analysis to avoid potential omitted variable bias, but results were excluded from the plot in panel b. All reported data are weighted considering the sampling design.

**Table A6 – Oaxaca-Blinder decomposition for differences in the treatment gap for depression (PHQ9 ≥ 10) according to race**

|  | **Treatment gap** | |
| --- | --- | --- |
|  |  |  |
| **Brown/Mixed + Black** | 0.7397*** |  |
| **White** | 0.6717*** |  |
|  |  |  |
| **Aggregate decomposition** | **Estimate** | **%** |
| **Difference** | 0.0679*** |  |
| **Explained** | 0.0376*** | 55.38% |
| **Unexplained** | 0.0304 | 44.77% |
|  |  |  |
| **Detailed decomposition** | **Explained** | |
|  | **Estimate** | **%** |
| **Sex** | -0.0007 | -1.03% |
| **Age** | -0.0080** | -11.78% |
| **Education** | -0.0029 | -4.27% |
| **Urban** | -0.0003 | -0.44% |
| **Region** | 0.0361*** | 53.17% |
| **Slum proxy** | -0.0008 | -1.18% |
| **Internet** | -0.0008 | -1.18% |
| **Employment status** | -0.0005 | -0.74% |
| **Income quintile** | 0.0225** | 33.14% |
| **Support of family and/or friends** | 0.0000 | 0.00% |
| **Lives with** | -0.0002 | -0.29% |
| **Participation in group, social and/or community activities** | -0.0027* | -3.98% |
| **Health insurance** | 0.0023 | 3.39% |
| **Registered with Family Health Team** | -0.0014 | -2.06% |
| **Violence** | -0.0022* | -3.24% |
| **Diagnosis of non-mental NCD** | 0.0007 | 1.03% |
| **Tobacco** | -0.0007 | -1.03% |
| **Physical activity** | -0.0004 | -0.59% |
| **Alcohol** | -0.0026 | -3.83% |

Note: the table shows full results from Oaxaca-Blinder decompositions of differences in the treatment gap for depression (PHQ9 ≥ 10) between individual self-identified as white and self-identified as brown/mixed or black. Results for categorical variables are the sum of the results of each category. All reported data are weighted considering the sampling design. * p-value < 0.1, ** p-value < 0.05, *** p-value < 0.01.

**Figure A8 – Oaxaca-Blinder decomposition for differences in the treatment gap for depression (PHQ9 ≥ 10 or currently treated) according to race**


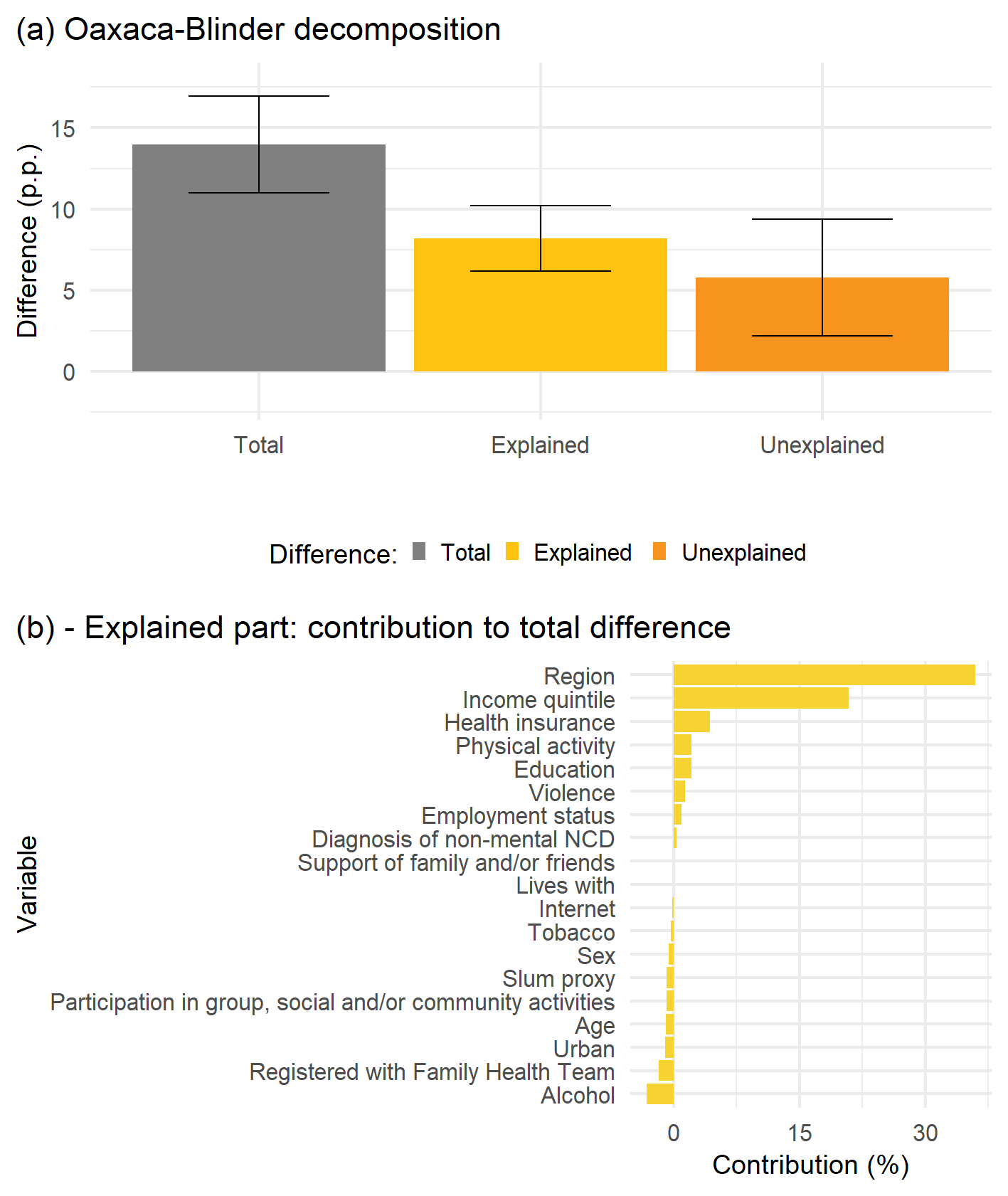


Note: the figure shows the treatment gap for depression grouping individuals with depression (PHQ9 ≥ 10) or currently treated according to racial/ethnic self-identification for White and Brown/mixed or Black individuals (panel a). Additionally, it shows results from an Oaxaca-Blinder decomposition of the differences in the treatment gap for depression between the two groups (panel b). Error bars display 95% confidence intervals. All reported data are weighted considering the sampling design.

**Figure A9 – Oaxaca-Blinder decomposition for differences in the treatment gap for depression (PHQ9 ≥ 10) according to race (excl. region)**


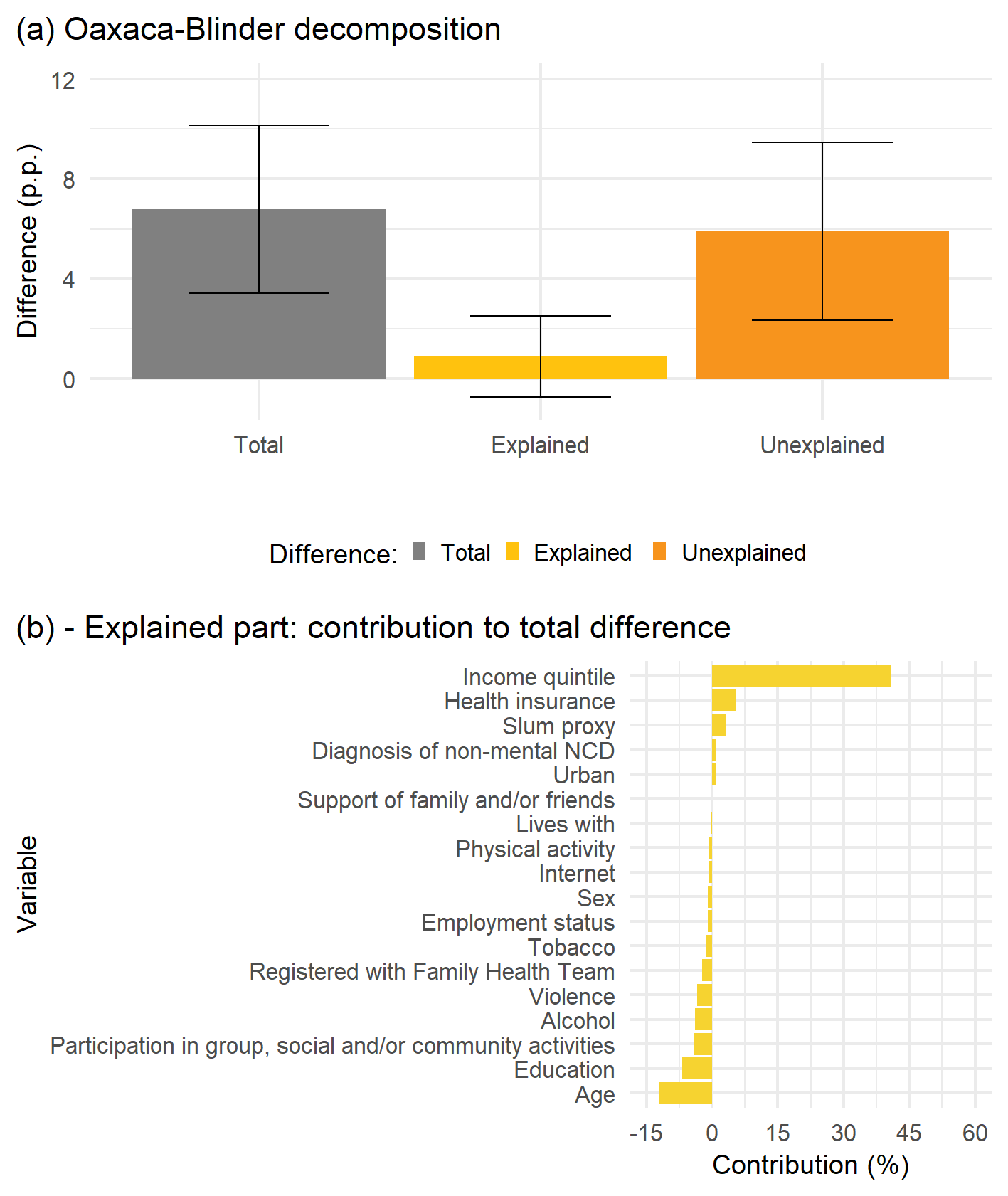


Note: the figure shows the treatment gap for depression according to racial/ethnic self-identification for White and Brown/mixed or Black individuals (panel a). Additionally, it shows results from an Oaxaca-Blinder decomposition of the differences in the treatment gap for depression (PHQ9 ≥ 10) between the two groups (panel b). Error bars display 95% confidence intervals. The analysis is similar to the one presented in Figure 4, but excluding region of residence from the analysis. All reported data are weighted considering the sampling design.

**Figure A10 – Oaxaca-Blinder decomposition for differences in the treatment gap for depression (PHQ9 ≥ 10) according to race (incl. rate of mental health professionals and HDI)**


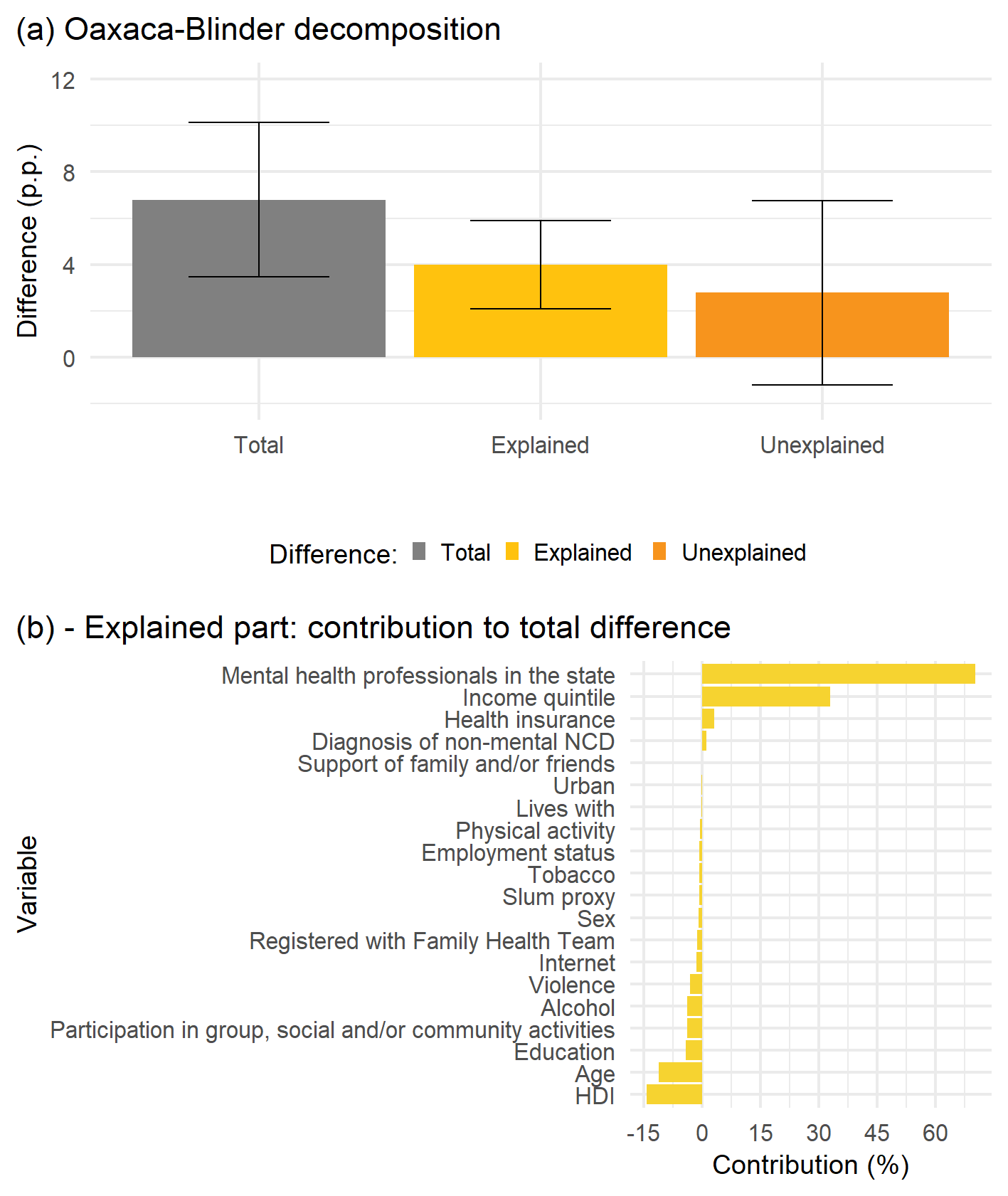


Note: the figure shows the treatment gap for depression (PHQ9 ≥ 10) according to racial/ethnic self-identification for White and Brown/mixed or Black individuals (panel a). Additionally, it shows results from an Oaxaca-Blinder decomposition of the differences in the treatment gap for depression between the two groups (panel b). Error bars display 95% confidence intervals. The analysis is similar to the one presented in Figure 4, but substituting region of residence for the rate of mental health professionals and HDI in the state of residence in the analysis. All reported data are weighted considering the sampling design.
